# Supplementary material for: Opposite macroevolutionary responses to environmental changes in grasses and insects during the Neogene grassland expansion
Source: Nat Commun. 2018 Nov 30;9:5089. doi: 10.1038/s41467-018-07537-8 (PMC6269479; doi:10.1038/s41467-018-07537-8)
Supplement: Supplementary file 1 — Supplementary Information [file 41467_2018_7537_MOESM1_ESM.pdf]

Supplementary Information for "Opposite macroevolutionary responses to environmental changes in grasses and insects during the Neogene grassland expansion"

Kergoat *et al.*

This PDF includes:

Supplementary Methods
Supplementary Figures 1 to 11
Supplementary Notes 1 to 2
Supplementary References

Supplementary Methods

Taxon sampling

Since 2004, Bruno Le Ru has conducted extensive fieldwork surveys to collect Sesamiina stemborers. Sampling trips were carried out in the following 17 sub-Saharan countries: Benin, Botswana, Burkina Faso, Cameroon, Democratic Republic of Congo, Eritrea, Ethiopia, Kenya, Lesotho, Madagascar, Mozambique, Republic of Congo, Republic of South Africa, Rwanda, Tanzania, Uganda and Zambia. Additional specimens were sampled in Ghana and Togo by Philippe Le Gall (IRD) and eight specimens from Swaziland and Zimbabwe were loaned by Martin Krüger (Transvaal Museum of South Africa; TMSA). Jérôme Barbut also conducted fieldwork in France and Portugal, mostly to collect Palearctic members of the closely related tribe Apameina and other noctuid species to be used as outgroups for the phylogenetic analyses. Regarding the latter we were careful to include several key genera (*Amphipyra*, *Apamea*, *Condica*, *Cryphia* and *Pyrrhia*) that were sampled in the study of Wahlberg *et al.*[1].

Both larvae and adults were collected. For larvae, sampling of visually damaged monocotyledonous plants (mostly Poales: grasses and sedges) was conducted to collect larval stages of noctuid stemborers within their wild host-plants ([2,3]; see also Supplementary Figure 11). In a given locality, though there are usually dozens of potential grass host-plants, but only a subset of them infested, and those usually attacked consistently by particular stemborer species (some localities were sampled several times at different seasons over several years, and many plant species were never found infested). Larvae were then reared on artificial diet until pupation and emergence of adults[4]. For adults, light traps set in open habitats were used in almost every locality. Over the years, a total of 80,000+ specimens was obtained, of which more than 52,000+ larvae were reared from 220 identified species of monocotyledon plants.

Most of the plant material was identified by Kathleen Gordon-Gray (Pietermaritzburg University, Republic of South Africa) and Simon Mathenge (Botany Department, University of Nairobi, Kenya).

Morphological studies

Morphological studies were carried out by Bruno Le Ru who is an expert on stemborer taxonomy and systematics. Both external and internal (i.e., genitalia) morphological characters were examined for species determination. Genitalia were dissected after immersion of the end of the abdomen in a boiling 10% potash bath for a few minutes, then cleaned, immersed in absolute alcohol for a few minutes and mounted on slides in Euparal (after separating the aedeagus from the rest of the genitalia in the male). Collected insects were identified by comparison with types and specimens housed in the Iziko Museums (Cape Town, South Africa), the Musée Royal d'Afrique Centrale (MRAC; Tervuren, Belgium), the Museo Civico di Storia Naturale di Milano (MCSN; Milan, Italy), the Muséum national d'Histoire naturelle (MNHN; Paris, France), the Natural History Museum (NHM; London, United Kingdom), the Swedish Museum of Natural History (NRM, Stockholm, Sweden) and the Transvaal Museum of South Africa (TMSA; Pretoria, South Africa).

All specimens were either assigned to a known species or assigned to putative morphospecies (with 'sp.' suffixes). It is noteworthy that several major taxonomic revisions (relying on integrative taxonomy approaches) are currently being developed; parts of these studies have been already published, such as for genera *Acrapex*[5,6,7,8], *Conicofrontia*[9] and *Sesamia*[10].

Molecular datasets

For this study, we first assembled a six locus molecular dataset for 1,393 specimens (specimen-level dataset; see Supplementary Data 1). Most individuals were extracted and sequenced by our team totaling 1,389 specimens, which represent 181 Sesamiina species, 36 Apameina species and 24 noctuid outgroups species. Sequences for the four remaining species were downloaded from GenBank (*i.e. Amphipyra pyramidoides* Guenée, *Condica videns* (Guenée), *Cryphia raptricula* (Denis & Schiffermüller) and *Pyrrhia exprimens* (Walker)) (see Supplementary Data 1 for accession numbers). For every DNA extraction, we only used a posterior leg to preserve most of the morphological integrity of the specimen; all corresponding voucher specimens are housed in the collection hosted in the IRD-CNRS UMR EGCE (Gif-sur-Yvette, France).

Total genomic DNA was extracted using the Qiagen DNeasy Kit (Hilden, Germany). Polymerase chain reaction (PCR) amplifications were conducted for four mitochondrial gene fragments: *cytochrome c oxidase subunit I* (*COI*; 657 bp), cytochrome b (*CYTB*; 991 bp), *ribosomal 12S* (*12S*; 381 bp), *ribosomal 16*S (*16S*; 547 bp). Two nuclear gene regions were also sequenced: *ribosomal 28S* (*28S*; 865 bp) and *elongation factor 1 al*pha (*EF1α*; 1,239 bp). We used the primers and settings detailed by Kergoat *et al.*[11]. Individual gene fragments were aligned using MAFFT 7 using the *Auto* option[12] and the reading frames of all protein-coding genes were checked using Mesquite 3.2[13]. The gene fragment alignments were then concatenated using Mesquite. All newly generated sequences were deposited on GenBank (accession numbers MH847792-MH853351).

Phylogenetic analyses

Phylogenetic analyses were conducted using maximum likelihood (ML). Bayesian inference (BI) analyses were only implemented for the species-level dataset because of convergence

issues with the larger specimen-level dataset (average split-frequencies of the runs above 5%). In both ML and BI, partitioned analyses were carried out to improve resolution[14]. For both datasets (specimen-level and species-level) partitions and substitution models were determined using PartitionFinder 1.1.1[15], with the *greedy* algorithm, the *unlinked* option for branch lengths, and the corresponding set of models (*beast*, *mrbayes* or *raxml*). The corrected Akaike information criterion (AICc) was used as a metric for ML analyses whereas the Bayesian information criterion (BIC) was used for BI analyses[16].

To assess the monophyly of Sesamiina species, phylogenetic relationships for the specimen-level dataset were inferred with maximum likelihood using RAxML 8.2[17]. Based on the AICc results we used three partitions with either a General time reversible (GTR)+G+I model or a GTR+G model. The best-scoring ML tree was obtained using a heuristic search implementing 10 random-addition replicates. Clade support was approximated using a non-parametric bootstrap procedure (500 replicates were used). RAxML analyses were performed on the CIPRES Science Gateway 3.3[18]. Nodes supported by bootstrap support values (BS) $\geq$ 70% were considered strongly supported following Hillis and Bull[19]. The 245 putative species (i.e., morphospecies determined by Bruno Le Ru) were recovered monophyletic in the resulting tree (Supplementary Figure 1).

Based on the results of the specimen-level dataset, we generated a 245-terminal species-level dataset (including 181 Sesamiina species, 36 Apameina species and 28 noctuid outgroups species), to properly conduct all subsequent analyses (i.e., dating analyses, historical biogeography, character optimization and diversification analyses). To better assess the phylogenetic relationships and clade support of the species-level tree, in addition to RAxML, we carried out ML analyses with IQ-TREE 1.5.5[20], and BI analyses with MrBayes 3.2.6[21]. For the ML analyses under RAxML, we used three partitions based on the AICc results with either a General time reversible (GTR)+G+I model or a GTR+G model. The best

ML tree was obtained using a heuristic search implementing 100 random-addition replicates. Clade support was then assessed using a non-parametric bootstrap procedure (500 replicates were used). For the IQ-TREE analyses under IQ-TREE, the same partitions were used, and the runs were performed using a dedicated web server[22]. IQ-TREE searches were conducted with default settings (*auto* substitution model option) and clade support assessed using 1,000 ultrafast bootstrap replicates[23]. Instead of using the standard threshold of 70% for BS, a higher threshold of 95% was used for ultrafast bootstrap support values (uBS), following authors' recommendations (http://www.iqtree.org/doc/Frequently-Asked-Questions#how-do-i-interpret-ultrafast-bootstrap-ufboot-support-values). The MrBayes analyses were performed with two partitions based on the BIC results. We conducted two independent runs with eight Markov chain Monte Carlo (MCMC): one cold and seven incrementally heated that ran for 50 million generations with trees sampled every 5,000 generations. MrBayes analyses were performed on the CIPRES Science Gateway 3.3[18] using BEAGLE to improve and speed up the likelihood calculation[24]. A conservative burn-in of 25% was then applied after checking for stability on the log-likelihood curves and the split-frequencies of the runs. Support of nodes for MrBayes analyses was provided by clade posterior probabilities (PP) as directly estimated from the majority-rule consensus topology. Nodes supported by PP $\geq$ 0.95 were considered strongly supported following Erixon *et al.*[25].

Bayesian divergence times estimation

Divergence times were estimated using Bayesian relaxed clocks as implemented in BEAST 1.8.2[26]. The partitions and substitution models were selected under PartitionFinder 1.1.1 following the settings above but with the *beast* set of models. We thus unlinked the partitions for the substitution models, but we linked the partitions for the clock models such that we worked with two molecular clocks (one for the mitochondrial compartment and one for the

nuclear part). We assigned a Bayesian lognormal relaxed clock with uncorrelated rates to each clock model[27]. The *Tree Model* was set to either a birth-death process or a Yule model[28].

In order to improve the convergence of runs we fixed the topology as inferred by the analysis of the species-level dataset with RAxML where 78% of the nodes are well-supported within the Sesamiina (70% when considering the whole tree). Therefore in the *Operators* panel of BEAUti, we unchecked the tree operators that usually modify the topology to ensure a fixed topology and a better convergence for the estimates of divergence times.

The fossil record of Lepidoptera is scarce, and the family Noctuidae is no exception to this pattern[29]. Indeed, only two fossils are considered to belong to Noctuidae, although these are ambiguous[29]. Besides, these two fossils are too young (late Pleistocene) to be useful for molecular dating of an old group like the Noctuidae[1]. Since there is no available described fossil for the tribe Apameini or closely related tribes, we relied on secondary calibrations derived from the comprehensive study of Wahlberg *et al.*[1] on Lepidoptera. In this study the authors used multiple fossil calibrations to infer divergence time estimates within Lepidoptera based on the phylogenetic dataset developed by Mutanen *et al.*[30]. We chose to constrain nodes in our tree that are shared with the tree of Wahlberg *et al.*[1]. We thus constrained the root as well as the stems of the genera *Condica* and *Cryphia* with uniform distributions encompassing the 95% credibility intervals estimated in Wahlberg *et al.*[1]. Note that the age estimates of these nodes largely overlap with the age estimates recovered in a previous study including the tribe Apameini (both Apameina and Sesamiina) and closely related lineages[31].

We designed two analyses by using either a birth-death process or a Yule model for the branching process prior in combination with a uniform prior distribution for the secondary calibrations. We then compared the fit of each analysis by performing a marginal likelihood (MLE) estimation using stepping-stone sampling[32] for each run, using 100 path

steps, and chains running for 500,000 generation with a log likelihood sampling every 5,000 cycles. The convergence of the runs was investigated using ESS.

BEAST analyses consisted of 50 million generations of MCMC with the parameters and trees sampled every 5,000 generations. A burn-in of 25% was applied after checking the log-likelihood curves. The maximum credibility trees, median ages and their 95% highest posterior density (HPD) were generated with TreeAnnotator 1.8.2[26]. BEAST analyses were performed on the CIPRES Science Gateway 3.3[18] using BEAGLE to improve and speed up the likelihood calculation[24].

Estimation of ancestral character states: Host-plant associations and ecological preferences

Host-plant associations were categorized at the family level for plants belonging to Cyperaceae and Typhaceae, the monophyly of which are not in question. To improve resolution of our analyses, and because Poaceae is the dominant host-plant family of Sesamiina, we coded Poaceae at the subfamily level. Host-plant associations were thus scored with the following six states: (i) Cyperaceae, (ii) Typhaceae, (iii) Poaceae: Arundinoideae, (iv) Poaceae: Chloridoideae, (v) Poaceae: Panicoideae, and (vi) Poaceae: Pooideae. A lower taxonomic level for the coding of Poaceae (such as tribes) was not envisioned as host-plants have not been confidently assigned to a specific genus for several stemborer species (see Supplementary Data 2). In addition, because our knowledge of the full host-range of each sesamiine species is likely incomplete, using a finer-scale level of character coding could potentially introduce biases in the corresponding analyses. Of 181 Sesamiina, we were unable to assign a host-plant state for seven; these were coded as unknown. The few species that use multiple host families/subfamilies (10 species feed on several family and eight species feed on several Poaceae subfamilies) were coded as polymorphic with multistate characters.

To account for multistate characters we analyzed the host-plant data under the dispersal-extinction-cladogenesis (DEC) model[33], as implemented in the ML framework of Lagrange C++[34]. We preferred the DEC model over the DEC+J model (as implemented in BioGeoBEARS[35]), because the latter often infers null or extremely low extinction rates[36], an effect of the model favoring direct dispersal over widespread ranges, which might not be adequate for reconstructing the history of ancient groups (founder-event speciation is more appropriate for island clades[35]). This argument is valid for any ancestral state estimation. Ancestral states for host-plant association were reconstructed using a simple unconstrained transition matrix (one rate for all transitions between states) allowing any host shift to be equally probable. To estimate the support of a given character state relative to another, the more likely state was selected according to a decision threshold, such that if the log-likelihoods between two states differ by two log-likelihood units, the one with the lower likelihood score is rejected.

The best ML tree obtained under RAxML was used in all ASE analyses. This tree was modified under Mesquite (*Prune clade* tool), by removing all species outside the Sesamiina; this in no way altered or mis-represented relationships within the subtribe. We also carried out an analysis of a truncated data set removing all Sesamiina species without genus- or species-specific host-plant data. The results support the same pattern of conserved of host-use on Panicoideae (Supplementary Figure 6). The results of ASE analyses allowed us to discuss the relevancy of the oscillation hypothesis (OH) and musical chairs hypothesis (MCH) in the context of Sesamiina diversification. Here we would like to emphasis that the taxonomic level (family/subfamily) used in our ASE analyses is similar with the one used in the reference studies on OH and MCH (see the introduction in the main text).

We also conducted two supplementary analyses with binary traits: In the first we explored the evolution of host-plant preference in relation to photosynthetic pathways (C_3 or

C_4); in the second we inferred the evolution of ecological preferences in relation to dry versus wet environments. To determine the photosynthetic pathways of plants we relied on several studies[37,38,39,40,41]. Preferences for dry or wet habitats were based on the fieldwork surveys conducted by Bruno Le Ru, in which habitats are classified following White[42] (see e.g., ref. [10]). Because of the binary nature of these two traits, ancestral character state estimations were carried out under ML using a one-parameter Markov k-state model with symmetrical rates[43], as implemented in Mesquite. We also used the same pruned ML tree as the guide tree (see above). The support of one state versus another (at a given node) was considered significant if the difference between their log-likelihoods was greater than or equal to 2.0[44].

Dated phylogeny and historical biogeography of the Panicoideae

The Sesamiina originated in the Afrotropics ca. 21 Ma[31] and feed primarily on panicoid grasses (Supplementary Data 2). Based on the results of optimization analyses described above, we had concluded that Sesamiina are phylogenetically strongly constrained on feeding on the subfamily Panicoideae (see the results of the multistate ASE), and this led us to investigate the pattern of diversification of Panicoideae. We obtained the dated phylogeny of Panicoideae from the global dated phylogeny of Poaceae[45] (stored in the *Dryad Respository* at: http://datadryad.org/resource/doi:10.5061/dryad.74b5d/5). Spriggs *et al.*[45] have estimated divergence times in Poaceae using two sets of fossil calibrations: the first includes only macrofossils (*Hypothesis 1*) and the second includes macrofossils plus phytoliths (*Hypothesis 2*). Phytoliths are rigid, microscopic structures made of silica, found in some plant tissues and persisting after the decay of the plant. Phytolith characters are usually phylogenetically unreliable[46] and their use in a molecular dating analysis is controversial[47], making the dating based on *Hypothesis 2* less conservative than that based on *Hypothesis 1*. As underlined in the main text of this study, age estimates for C_4 grass lineages inferred under *Hypothesis 2* are

also questionable because they significantly predate the fossil record of open grasslands. For this study, we selected the dated tree as estimated by *Hypothesis 1* (only macrofossils). The *Prune clade* tool of Mesquite was used to isolate the Panicoideae from the rest of dated tree of the Poaceae; the resulting pruned tree contains 805 species out of 3,500 known species.

We inferred global historical biogeography of Panicoideae using the DEC model[33] as implemented in the ML framework of Lagrange C++[34]. Global species distributions were divided into seven categories: WP = Western Palearctic (Europe), EP = Eastern Palearctic (Asia), NEA = Nearctic (North America), NT = Neotropics (South and Central America, and the Caribbean Islands), AF = Afrotropics, OR = Oriental (India and Indomalayan Archipelago), and AU = Australasia. For each biogeographic region, we categorized the presence or absence of the 805 species included in the dated tree of the Panicoideae (Supplementary Data 3). The occurrence data were compiled from different sources in the literature[48,49,50,51,52] and through the use of online databases, such as the global invasive species database (GISD; http://www.iucngisd.org/gisd/), the FAO database (http://www.fao.org/ag/agp/AGPC/doc/Gbase/), the Kew Royal botanic garden database (https://www.kew.org/data/grasses-db.html), the IUCN red list (http://www.iucnredlist.org/), the Tropicos database (http://www.tropicos.org/), the USDA database (https://plants.usda.gov/java/). We also used the global biodiversity information facility (GBIF; https://demo.gbif.org/) where we systematically checked for the most ancient records to better infer native ranges (tentatively limiting biases linked to intentional or unintentional introductions). Following the views of several authors[53,54,55], taxa that overlapped a given area only marginally were not assigned to it. The species with the largest current native range is assigned four of the seven areas. Consequently, we set a maximum of four areas per ancestral range at each node. While DEC allows the building of dispersal rate matrices, we did not use them because grasses are very good dispersers (for example, by wind[56]) with

dynamic biogeographical patterns[57]. Because the log-likelihood of a time-stratified biogeographical DEC model was substantially lower than those of the DEC model without time slices (log-L $_{\text{unconstrained DEC}}$ = -1755.97 vs. log-L $_{\text{time-stratified DEC}}$ = -2123.45), we did not used a time-stratified biogeographical model. Therefore, the historical biogeography of Panicoideae was estimated with an unconstrained DEC model with only one time slice (Supplementary Figure 7). Our aim was not to study the fine-scale history of geographic diversification, but rather to identify when Panicoideae colonized the Afrotropics and whether this estimate coincided with the temporal Afrotropical origin of Sesamiina.

Diversification of both clades

We wished to investigate whether the modes of species diversification were similar between the Sesamiina and their ancestral host-plants, the Panicoideae, and to this end we used a stepwise procedure that took the best time-calibrated phylogenies as the basis for the analyses. When it was possible to run the models on multiple phylogenetic trees we took into account age uncertainties by sampling randomly 100 dated trees from the posterior distribution of the BEAST dating analyses. Unfortunately, for the Panicoideae, we only had the consensus tree and therefore could not address the effect of age uncertainties on the inference of diversification.

The procedure was as follows: (i) using three complementary approaches (BAMM 2.5[58]; RPANDA 1.3[59]; TreePar 3.3[60]) we first estimated whether the clade diversification deviated from a constant birth-death model and if shifts in speciation and extinction rates occurred; (ii) we then tested if past temperature changes had similar impacts on the diversification of both clades; (iii) we tested whether diversification had been influenced by the role of fluctuations of atmospheric carbon concentrations for plants, while simultaneously testing the role of grassland expansion through time for the Sesamiina; and (iv) we tested the

role of diversity-dependence processes on the diversification of the moths only (not on the plants because we had found that speciation rates increased through time, which contradicts diversity-dependence).

First, we used BAMM 2.5 to estimate speciation and extinction rates through time and among/within clades[58]. BAMM was constructed to study complex evolutionary processes on phylogenetic trees, potentially shaped by a heterogeneous mixture of distinct evolutionary dynamics of speciation and extinction across clades. BAMM can automatically detect rate shifts and sample distinct evolutionary dynamics that explain the diversification dynamics of a clade without a priori hypotheses on how many and where these shifts might occur. Evolutionary dynamics can involve time-variable diversification rates; BAMM allows for speciation to vary exponentially through time while extinction is maintained at a constant rate: clades are allowed to vary independently in rates of diversification, which is useful for testing the hypotheses of diversification shifts associated with putative key innovations.

We ran BAMM by setting four MCMC running for 20 million generations and sampled every 2,000 generations. In this procedure, a compound Poisson process is implemented for the prior probability of a rate shift along any branch. We used a gradient of prior values ranging from 0.1 to 10 to test the sensitivity to the prior, thereby taking into consideration recent concerns raised on the reliability of BAMM estimates[61], but see ref. [62]. For the stemborer moths, we set a global sampling fraction for the tree ($f = 0.905$) because we cannot assign missing species to specific clades even with morphological data (given the polyphyletic nature of several genera such as *Acrapex* and *Sesamia*). For the panicoid grasses, we set clade-specific sampling fractions for each tribe based on the most recent taxonomic estimates (see ref. [45]). We performed independent runs (with a 15% burn-in) using different seeds to assess the convergence of the runs with effective sample size. The MCMC output data was processed using BAMMtools 2.1.6[63] by estimating (i) mean global rates of

diversification through time, (ii) the configuration of diversification rate shifts evaluating alternative diversification models as compared by posterior probabilities, and (iii) clade-specific rates through time whenever a distinct macroevolutionary regime is identified.

Second, the ML approach of Morlon *et al.*[64] was used to corroborate the BAMM results. This approach is a birth-death method that extends previous birth-death methods by liberalizing the variability of speciation and/or extinction rates through time, and subclades may have different speciation and extinction rates. This method reduces assumptions of extinction rate constancy (unlike BAMM[58]), and allows negative diversification rates in clades where diversity decreases because extinction exceeds speciation[64]. We identified six nested diversification models to test with this approach: (i) a Yule model, where speciation is constant and extinction is null; (ii) a constant birth-death model, where speciation and extinction rates are constant; (iii) a variable speciation rate model without extinction; (iv) a variable speciation rate model with constant extinction; (v) a rate-constant speciation and variable extinction rate model; and (vi) a model in which both speciation and extinction rates vary. Models were compared by computing the ML score of each model and the resulting AICc[65].

Third, we estimated whether potential shifts in speciation and extinction rates occurred in the phylogeny using TreePar[60] and the function 'bd.shifts.optim'. We compared several birth-death models including zero (constant-rate model) to four diversification rate shifts during the lineage evolution while taking into account age uncertainties (100 randomly selected posterior trees from the BEAST posterior distribution). All analyses were carried out with the following non-default settings: taxon sampling set to 181/200, start = 0, end = crown age, and grid = 0.1 million years (Myr) for a fine-scale estimation of rate shifts. We calculated AICc scores and computed Likelihood Ratio Tests (LRT) to select the best-fit

between the different models allowing incrementally more shifts during the evolution of the clade.

We further tested the impact of past environmental changes on the diversification of both clades to assess whether they responded similarly to changing abiotic conditions. An environmental-dependent model was used in which speciation and/or extinction could vary as a function of temporal variation of the environment[66]. Thus, for the paleoenvironment-dependent models, we needed to incorporate the temporal variations of a given environmental proxy (e.g., temperature through time). Here we compiled paleodata for variables associated with macroevolutionary hypotheses of diversification and that best represent paleoclimatic changes: temperature data (inferred from $\delta^{18}O$ measurements) taken from Zachos *et al.*[67] (see also Figure 2d), atmospheric CO_2 data retrieved from Beerling & Royer[68] (see also Figure 2f, red curve), and $\delta^{13}C_{organic}$ for organic carbon sequestration[69,70] (see also Figure 2f, black curve). Temperature is considered an important driver of biodiversity patterns and evolutionary processes[71], and therefore, we tested the role of warming and cooling events on the diversification of both clades. Levels of atmospheric CO_2 are thought to impact photosynthetic organisms[72,73,74,75], and were therefore used to test their impact on the diversification of plants. The $\delta^{13}C_{organic}$, estimated from tooth enamel, reflects global changes in organic carbon sequestration[69,70]. In the Cenozoic, $\delta^{13}C$ is the best proxy for reconstructing ancient C_4 grasslands since it represents the proportion of C_4 and C_3 grasses[70,73,76], and is therefore likely to be crucial to the evolution of both grasses and the herbivores that feed on them. To confirm the effect C_4 grasslands had on moth diversification, we also performed additional diversification analyses using the recently estimated proportion of C_4 plants in eastern Africa through time[77] instead of the global $\delta^{13}C_{organic}$. Using the data and phylogenies for both clades, we compared constant-rate models, time-dependent models (see above), and paleoenvironment-dependent models in a single framework using AICc. For stemborer moths

we fitted paleoenvironment-dependent models of diversification in which speciation and/or extinction may vary according to a dependence with temperature and $\delta^{13}C_{organic}$. For Panicoideae grasses we fitted models with temperature- and CO_2-dependence.

We also tested the hypothesis that diversity is bounded or at equilibrium, meaning that diversity expanded rapidly in early diversification, occupying available extrinsic niches early and appearing saturated toward the present. We explored the effect of diversity-dependence on speciation and extinction rates using the method of Etienne *et al.*[78] implemented in the R-package DDD 2.7, applying five different models: (i) speciation is a linear function of diversity, without extinction; (ii) speciation is a linear function off diversity, with extinction; (iii) speciation depends exponentially on diversity, with extinction; (iv) speciation does not depend on diversity and extinction is a linear function of diversity and; (v) speciation does not depend on diversity and extinction depends exponentially on diversity. For each model, the initial carrying capacity was set to the current number of described species. We did not perform DDD analyses on panicoid grasses (or on the subclades) because the speciation rates of all groups increase through time and thus contradict the expected pattern under the hypothesis of diversity dependence.

Finally we also performed supplementary analyses using the Binary State Speciation Extinction (BiSSE) model[79] to estimate whether the respective diversification rates of Afrotropical and non-Afrotropical panicoid species are comparable. To do so, first we estimated the number of native Afrotropical panicoid species. We determined the number of African panicoid species by extracting species distributions from the GRASSWORLD database (http://grassworld.myspecies.info/en), using its curated checklist of African grasses (http://grassworld.myspecies.info/sites/grassworld.myspecies.info/files/Africa.doc). An estimated total of 1,131 African panicoid species was determined using this checklist in combination with the list of panicoid genera published in the worldwide phylogenetic

classification of the Poaceae by Soreng *et al.*[80], which also relies on the GRASSWORLD database. Although the African GRASSWORLD species checklist aims at only including native species it is still a work in progress so we used additional filters to better account for the occurrence of non-native species. First, we removed the 29 species belonging to genera that are known to be non-native to the Afrotropical region[80]. Second, as a way to detect potentially non-native species, we removed the 67 most widespread species (hereby defined as distributed in more than 20 African countries). Finally we removed seven species that were exclusively found in North Africa (which is part of the Palearctic biogeographic realm). As a result we ended up with an estimate of 1,034 native panicoid species for the Afrotropical region, to be compared with a total species richness of 3,560 species for the whole subfamily[80]. Because several non-native species are still potentially included in our list (native distributions are sometime ambiguous) we think that the number of native panicoid species is actually comprised between 900 (conservative low boundary) and 1,034 species. We then carried out eight BiSSE models, from the null model (no variation of rates) to the most complex model in which all parameters of speciation, extinction and transition are estimated. We considered a conservative Afrotropical species number sampled in the phylogeny (i.e., 155 species) by excluding all species not exclusively distributed in the Afrotropics. The most complex model (*all.free*) was defined as the best-fit model by the AICc comparisons (see the table below). Whatever the total Afrotropical diversity estimates are (900 vs. 1034), for both groups we found very similar net diversification rates (r = *lambda* - *mu*) comprised between 0.24 and 0.28 events/Myr/lineage (0.24-0.25 for the Afrotropical native Panicoideae, vs. 0.28 for the remaining panicoid species). These analyses support the hypothesis that the Afrotropical diversity of Panicoideae increased at the same pace as the overall diversity of other members of the subfamily.

BiSSE analyses with an estimated diversity of 900 species for the Afrotropical panicoid species							
Model	NP	logL	AICc	lambda0	lambda1	mu0	mu1
Null model	3	-2355.263	4716.556	NA	0.7692	NA	0.5099
lambda.free	4	-2346.041	4700.131	0.7454	0.5909	NA	0.414
mu.free	4	-2345.351	4698.751	NA	0.7192	0.3891	0.5523
q.free	4	-2345.377	4698.804	NA	0.7734	NA	0.5154
lambda.mu.free	5	-2345.296	4700.668	0.7225	0.6876	0.3907	0.5193
lambda.q.free	5	-2341.706	4693.487	0.7546	0.6576	NA	0.4516
mu.q.free	5	-2342.989	4696.053	NA	0.7463	0.4482	0.5453
all.free	6	-2340.138	4692.382	0.8147	0.5028	0.5312	0.2454

BiSSE analyses with an estimated diversity of 1,034 species for the Afrotropical panicoid species							
Model	NP	logL	AICc	lambda0	lambda1	mu0	mu1
Null model	3	-2350.249	4706.527	NA	0.7544	NA	0.4924
lambda.free	4	-2343.462	4694.973	0.7348	0.6017	NA	0.409
mu.free	4	-2342.66	4693.37	NA	0.7097	0.3842	0.526
q.free	4	-2343.125	4694.3	NA	0.7593	NA	0.4991
lambda.mu.free	5	-2342.658	4695.391	0.7135	0.7102	0.3888	0.527
lambda.q.free	5	-2340.63	4691.336	0.7445	0.6627	NA	0.4453
mu.q.free	5	-2341.275	4692.625	NA	0.7317	0.4334	0.521
all.free	6	-2340.196	4692.498	0.7754	0.5745	0.4872	0.3309

To provide more support to this hypothesis regarding the similarity of the diversification dynamics of both Afrotropical and non-Afrotropical lineages, we also present several additional lines of evidence.

1. The first is related to the fact that speciation rates of both Afrotropical and non-Afrotropical lineages largely overlap as illustrated by the following output from BiSSE.

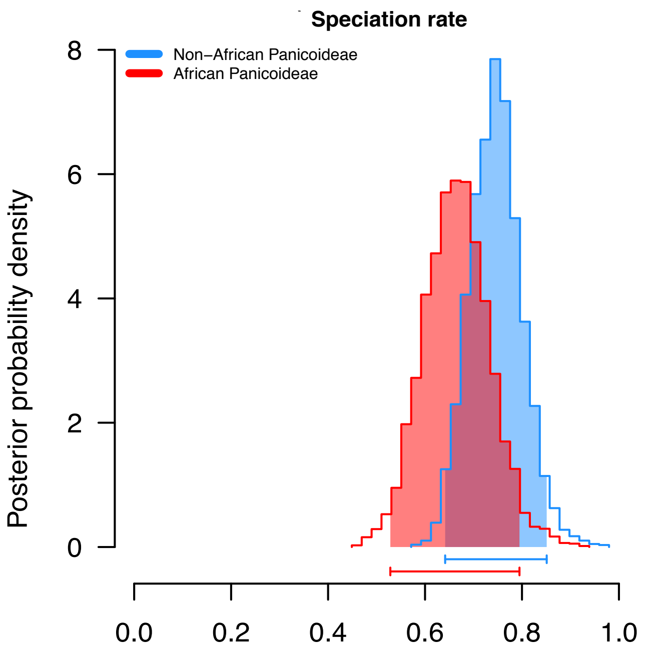

The fact that *within Afrotropical* or *within non-Afrotropical* lineages speciation rates are overlapping is also consistent with results of BAMM analyses that highlighted that Afrotropical and non-Afrotropical lineages have similar diversification regimes (please see below).

2. Another points worth underlining surrounds the results of BAMM analyses in the light of the BioGeoBEARS analyses. Three distinct net diversification rate (DR) regimes are inferred under BAMM (please see below, on the left). When visualizing these distinct DR regimes on the results of BioGeoBEARS analyses (please see below, on the right) two interesting conclusions can be made:

First, for the three distinct diversification regimes (labelled 1, 2 and 3) there is a mix of Afrotropical (with nodes circled in red on the tree at the right) and non-Afrotropical lineages (other variously coloured nodes of the same tree). Afrotropical lineages are definitely not associated exclusively with any one diversification regime. Here it is worth mentioning that the lack of Afrotropical lineages for the diversification regime labelled 2 (a subset of the Paspaleae) can be accounted for by the fact that Afrotropical Paspaleae lineages (Afrotropical *Axonopus, Hymenachne, Ichnanthus* and *Paspalum* spp. plus Afrotropical endemic Paspaleae genera such as *Baptorachis* or *Lecomtella*) are not represented in the phylogeny.

Second, the two shifts in diversification rates (leading to diversification regimes labelled 2 and 3) are not apparently associated with shifts in distribution. The first shift (labelled 2) occurs for a subset of an existing mostly Neotropical clade, and not linked to a change of distribution or biogeographic event (ancestral lineages before and after the DR shift stayed in the Neotropical region). Likewise, the second shift (labelled 3) occurs within a widespread group, and it is not linked to a change of distribution (ancestral lineages before and after the DR shift stayed in the Oriental region).

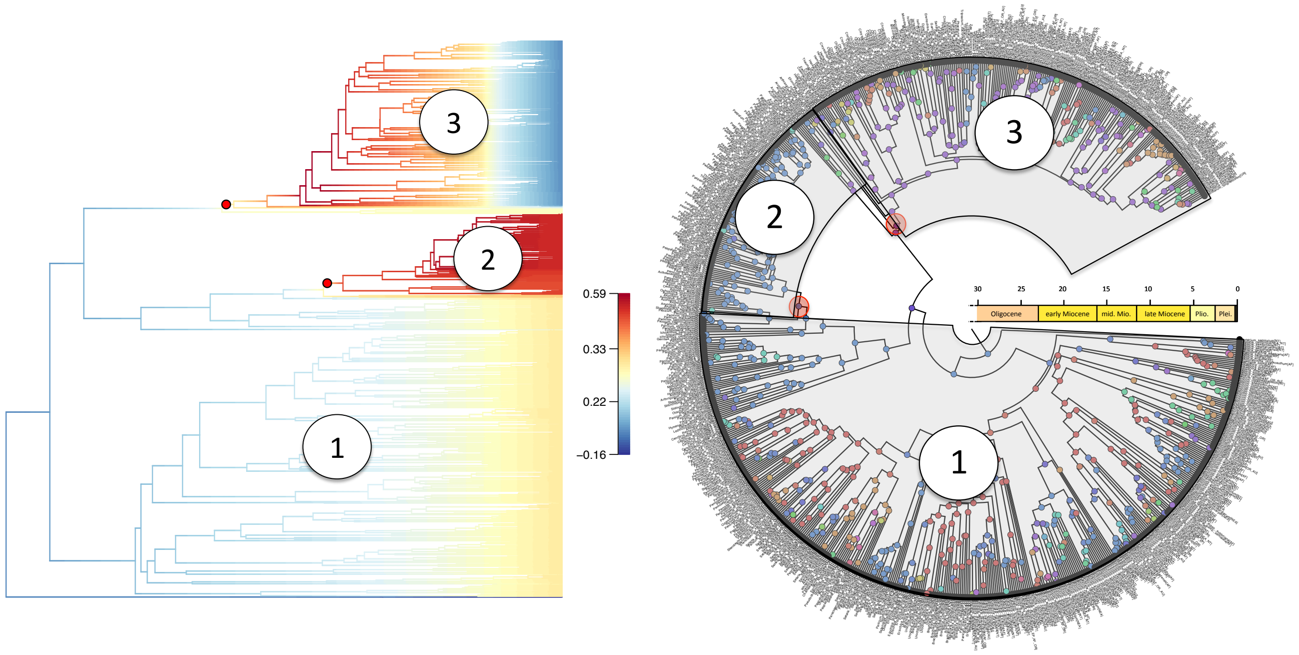

3. Finally we conducted additional analyses under BiSSE to infer the pattern of species accumulation through time. Based on the most likely BiSSE model, we generated lineages-through-time (LTT) plots for both Afrotropical and non-Afrotropical lineages. Based on the most likely BiSSE model, we generated lineages-through-time (LTT) plots using an estimated diversity of 900 and 1,034 Afrotropical panicoid species (Supplementary Figure 9a-b). Both Afrotropical and non-Afrotropical lineages show increasing patterns of species accumulation, with no distinct up-shifts and more importantly no plateau of diversity. It is also worth mentioning that the slopes would be steeper if we used a complete species-level phylogeny; though ca. 3,500 species panicoid are known, only 805 panicoid species were sampled in the Timetree from Spriggs et al.[45]. We also generated a LTT plot for the Sesamiina (Supplementary Figure 9c), which relied on a more comprehensive sampling for the stemborers (181 species to be compared with ca. 200 known species). It is worth underlining that due to differences in sampling completeness between the Panicoideae (805 sampled species vs. ca. 3,500 known species)

and the Sesamiina (181 sampled species vs. ca. 200 known species), it would be misleading to directly compare both LTT plots.

Overall, the extent of the analyses performed (BAMM, BiSSE and BioGeoBEARS) corroborate our conclusions that (i) diversification is similar between Afrotropical and non-Afrotropical lineages and (ii) changes in diversification rates through time are not restricted to a specific area (i.e., continent), and can thus be more readily attributed to trait acquisition or global abiotic events than to ecological opportunity mediated by the geographic colonization of a novel area (continent).

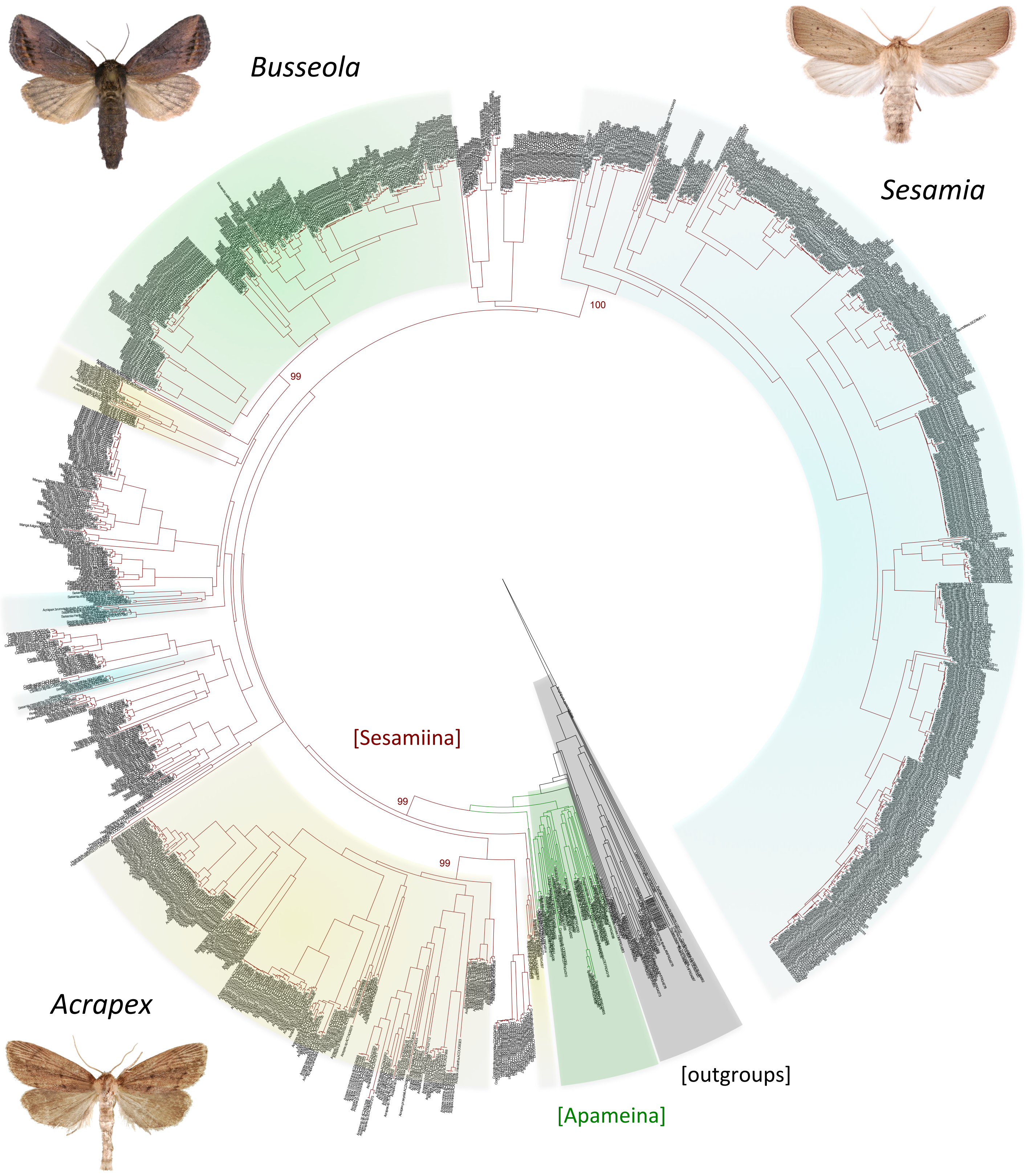

Supplementary Figure 1. Maximum likelihood tree for the moths (specimen-level dataset). Best-fit maximum likelihood tree (L = -127162.57) resulting from the analysis of the specimen-level dataset (245 species) carried out under RAxML. Bootstrap support values are only provided for major nodes. Noctuid outgroup taxa (38 individuals from 28 species) are highlighted using black branches and a grey filter while Apameina taxa (47 individuals from 36 species) are highlighted using green branches and a green filter. Sesamiina (1,308 individuals from 181 species) are highlighted using red branches. Within Sesamiina, the most diverse genera are highlighted using yellow, green and blue filters (for *Acrapex*, *Busseola* and *Sesamia*, respectively); please note that species with a sl. (sensu lato) tag are putatively assigned to extant genera pending the publication of ongoing comprehensive taxonomic revisions. Habitus of adults belonging to the species *Acrapex rubona* Le Ru, *Busseola fusca* (Fuller) and *Sesamia nonagrioides* (Lefèbvre) are presented for illustrative purpose. Copyright notes: all pictures were taken by B. Le Ru (last author of the paper).

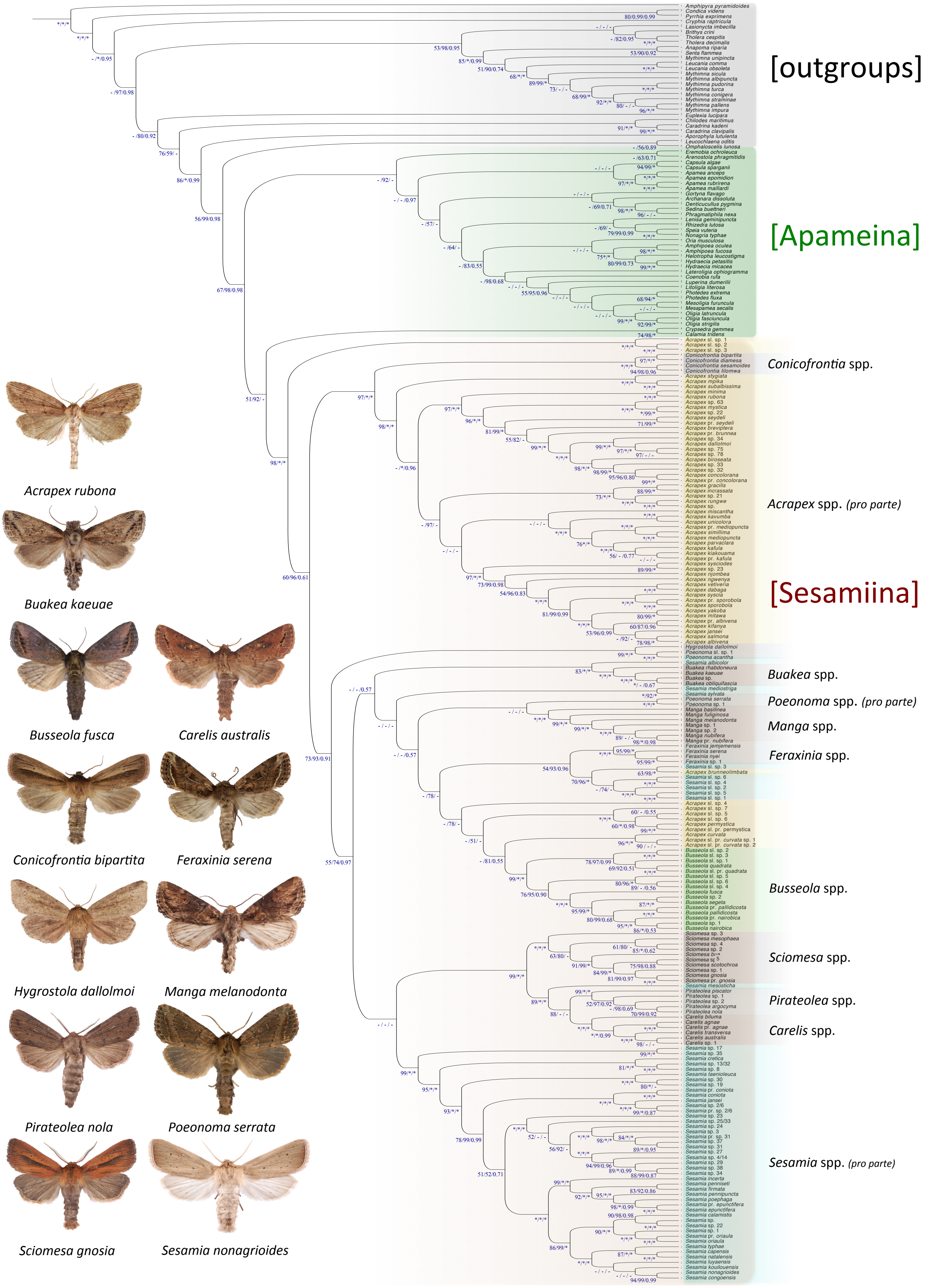

Supplementary Figure 2. Maximum likelihood tree for the moths (species-level dataset). Best-fit maximum likelihood tree (L = -88365.60) resulting from the analysis of the species-level dataset (245 species) carried out under RAxML. Support values are provided on nodes (BS from the RAxML analyses, uBS from the IQ-TREE analyses and PP from the MrBayes analyses, in that order). We used the abbreviation '-' either for low support values (below 50% for BS/uBS or below 0.50 for PP) or whenever a particular node was not recovered by a given method. Noctuid outgroups, Apameina and Sesamiina taxa are highlighted using either a grey, green, or orange filter (in that order). Within Sesamiina, distinct genera are highlighted using various filters; please note that species with a sl. (sensu lato) tag are putatively assigned to extant genera pending the publication of ongoing comprehensive taxonomic revisions. On the left, pictures of representatives from all Sesamiina genera are presented for illustrative purpose. Copyright notes: all pictures were taken by B. Le Ru (last author of the paper).

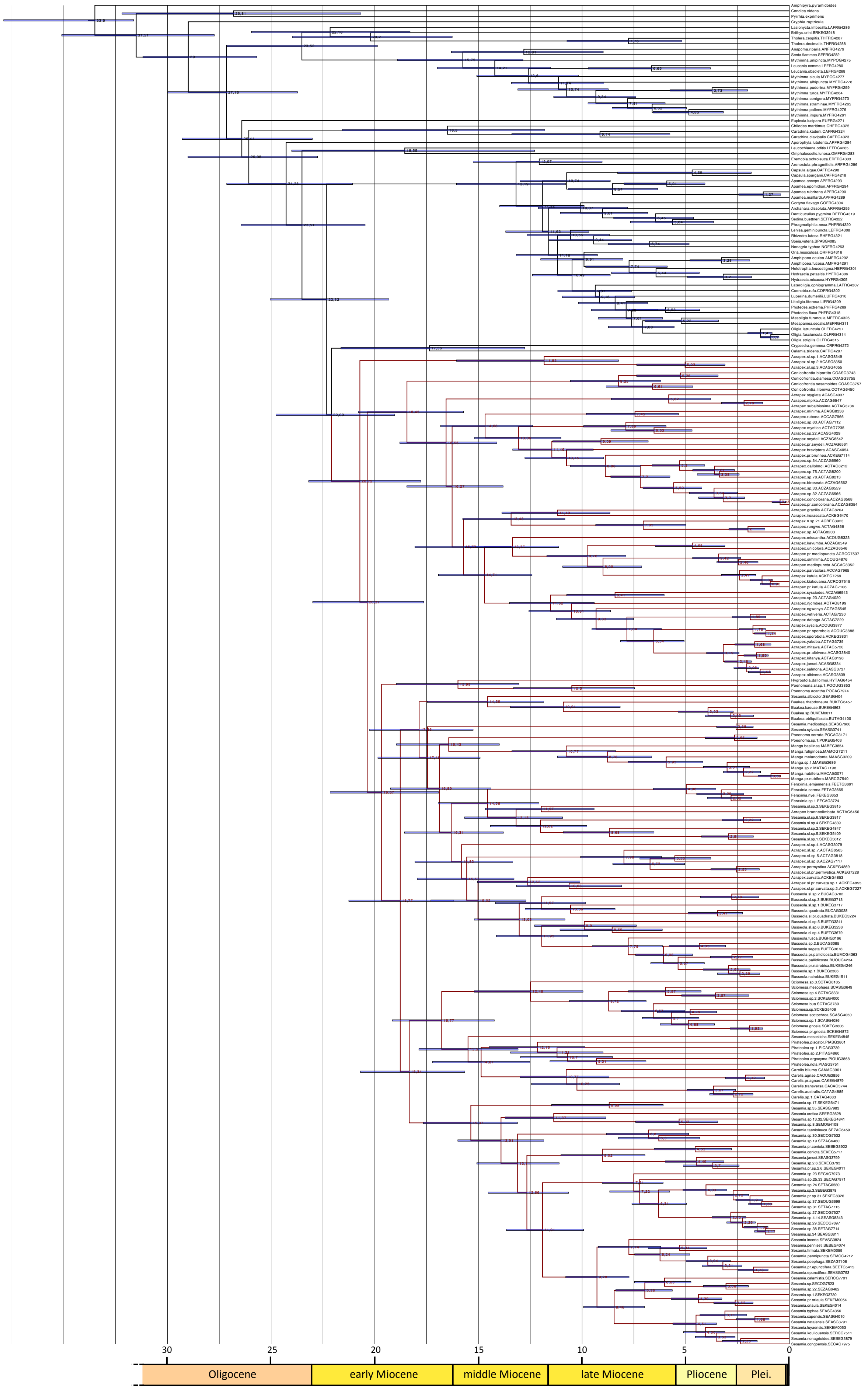

Supplementary Figure 3. Timetree for the moths with birth-death tree speciation priors. Dated phylogeny resulting from BEAST analyses of the species-level dataset, using birth-death tree speciation priors. Median age estimates are presented on nodes; horizontal bars on nodes are also used to provide the 95% highest posterior density interval of age estimates. Sesamiina species are highlighted using red branches.

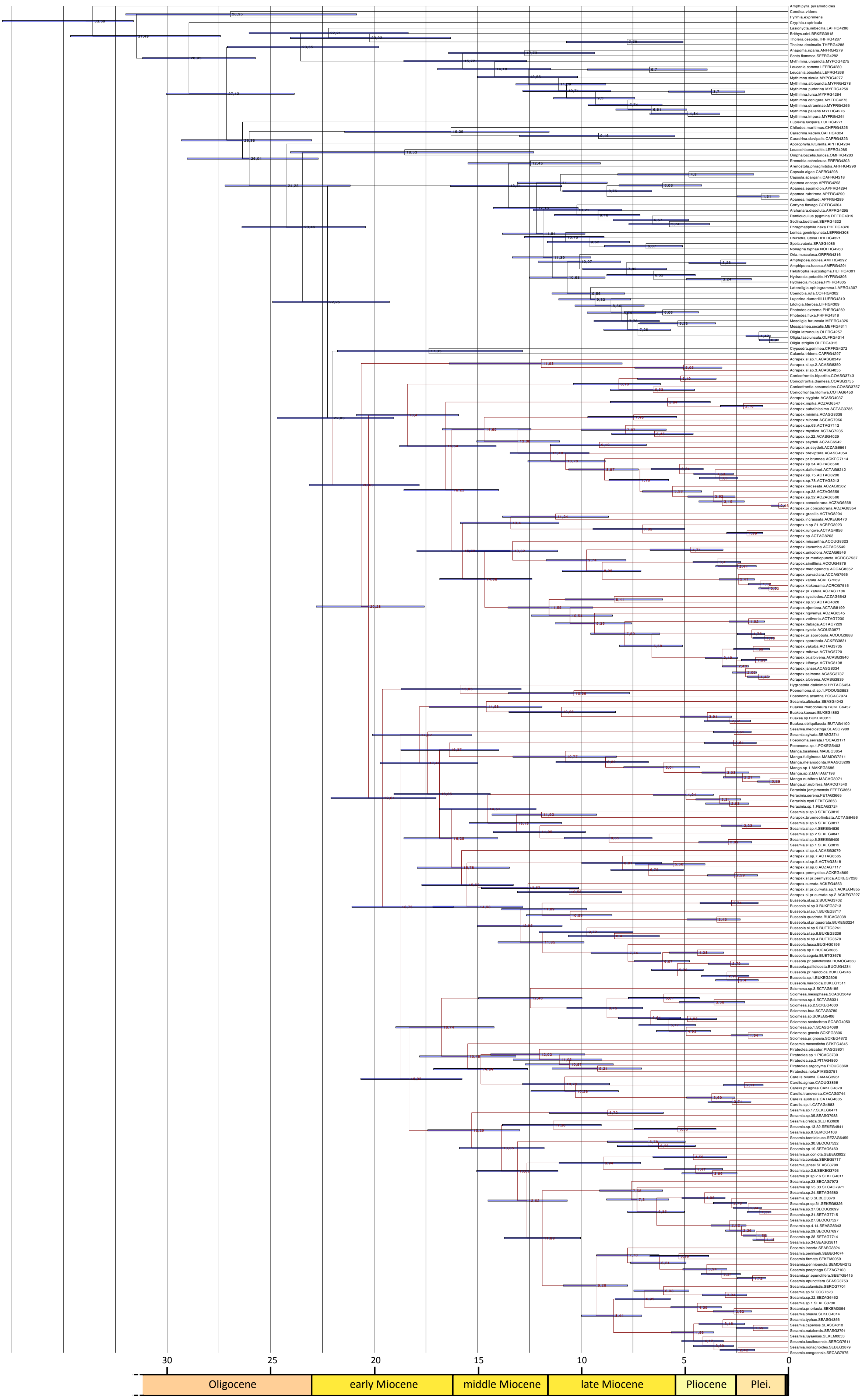

Supplementary Figure 4. Timetree for the moths with Yule tree speciation priors. Dated phylogeny resulting from BEAST analyses of the species-level dataset, using Yule tree speciation priors. Median age estimates are presented on nodes; horizontal bars on nodes are also used to provide the 95% highest posterior density interval of age estimates. Sesamiina species are highlighted using red branches.

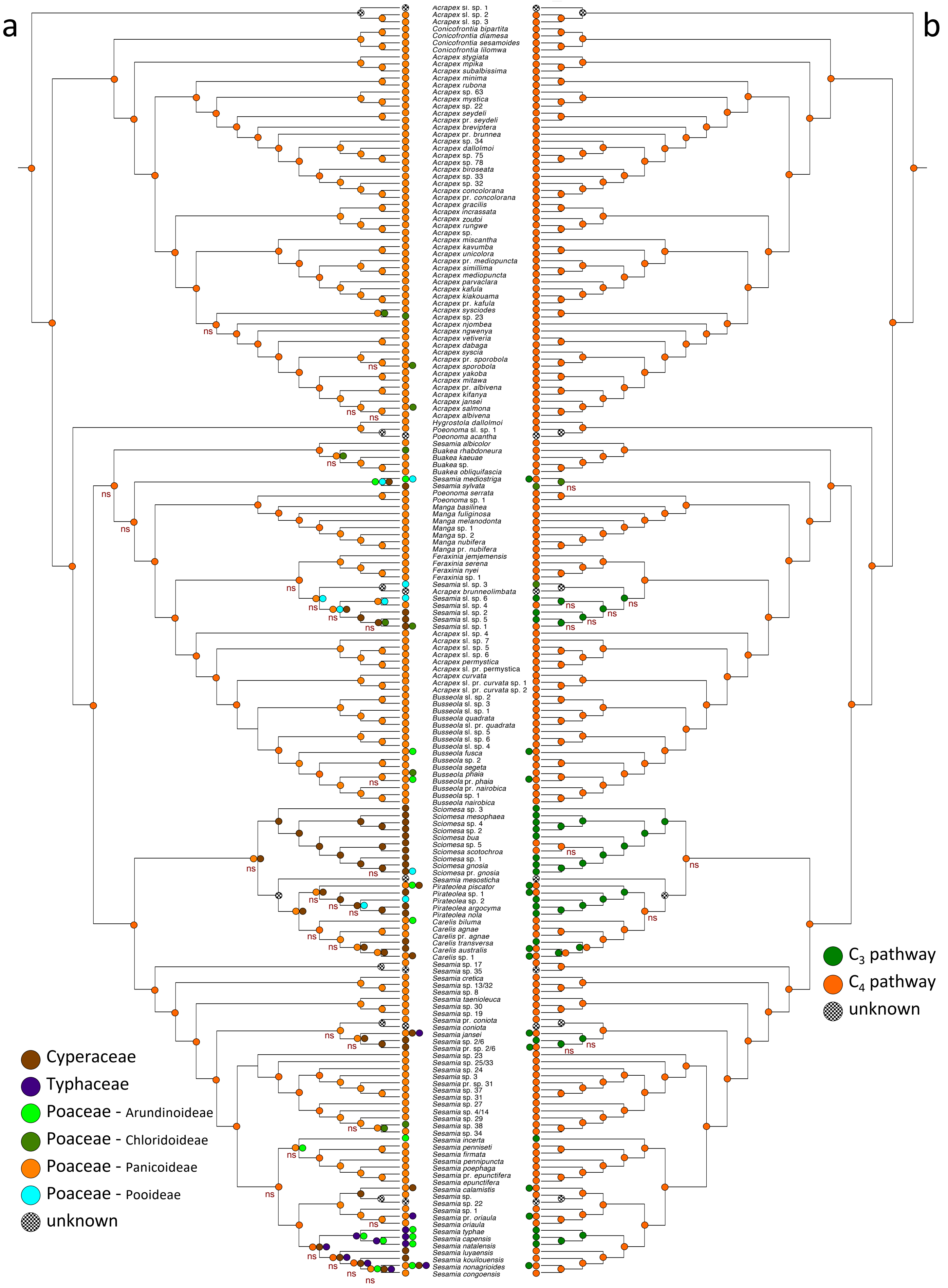

Supplementary Figure 5. Results of the character optimizations of host-plant associations. **(a)** On the left: results of the character optimization of host-plant associations, carried out under maximum likelihood with a DEC model. Host-plants were either categorized at the family level (for plants belonging to Cyperaceae and Typhaceae) or at the subfamily level (for plants belonging to Poaceae). **(b)** On the right: results of the character optimization of host-plant preferences, where host-plants were categorized based on the nature (C_3 or C_4) of their photosynthetic pathway. Corresponding analyses were carried out with Mesquite; for each node, the most likely character state (or combination of character states) is figured; if the corresponding character state (or combination of character states) is not statistically supported the abbreviation 'ns' (for 'not supported') is added.

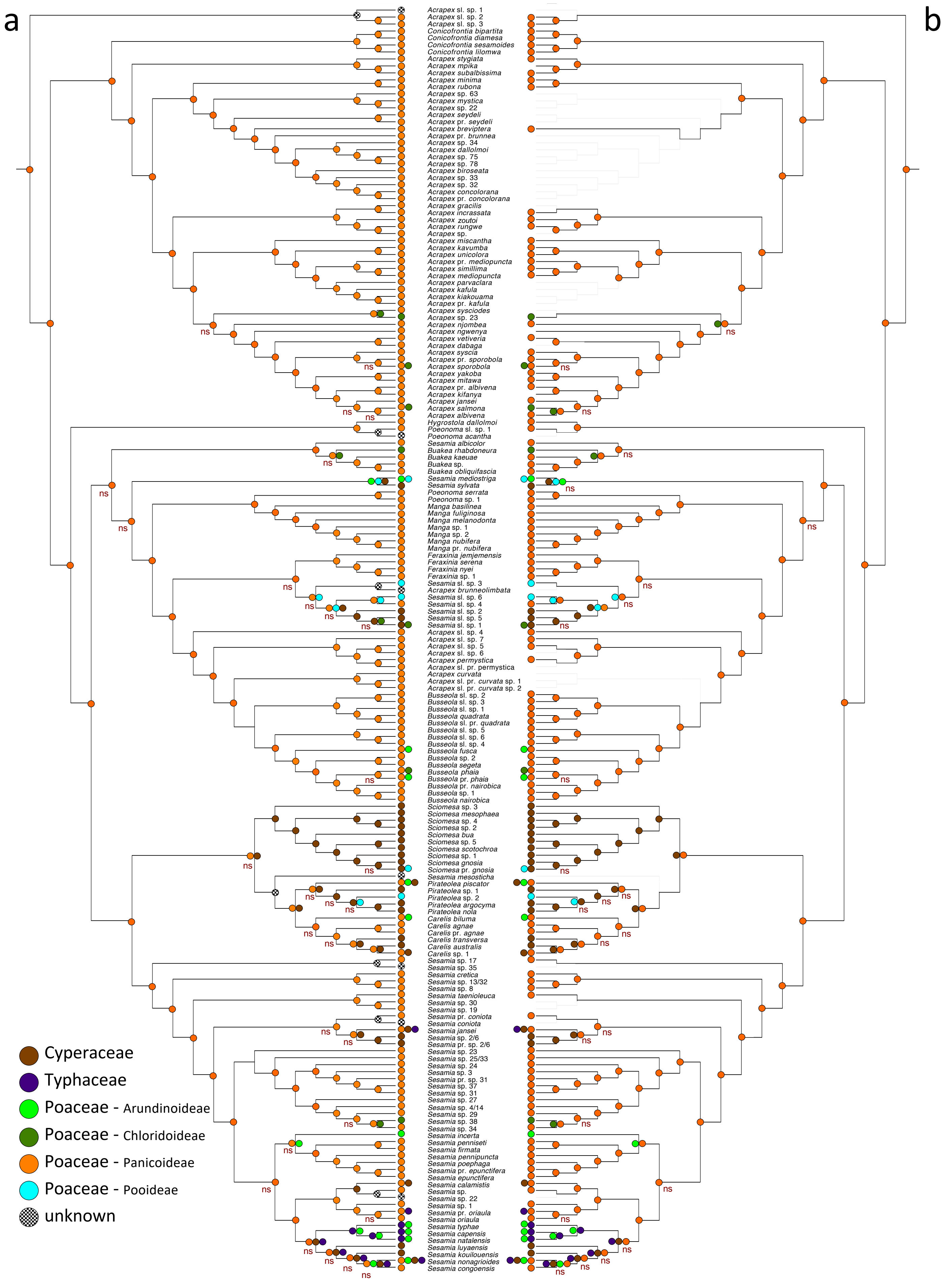

Supplementary Figure 6. Comparison of character optimizations of host-plant associations. **(a)** On the left: results of the character optimization of host-plant associations, with all host-plant data. **(b)** On the right: results of the character optimizations of host-plant associations, where only Sesamiina species with accurate host-plant assignations (genus or species level) are included. For both analyses, character optimizations of host-plant associations were carried out under maximum likelihood with a DEC model. Host-plants were either categorized at the family level (for plants belonging to Cyperaceae and Typhaceae) or at the subfamily level (for plants belonging to Poaceae).

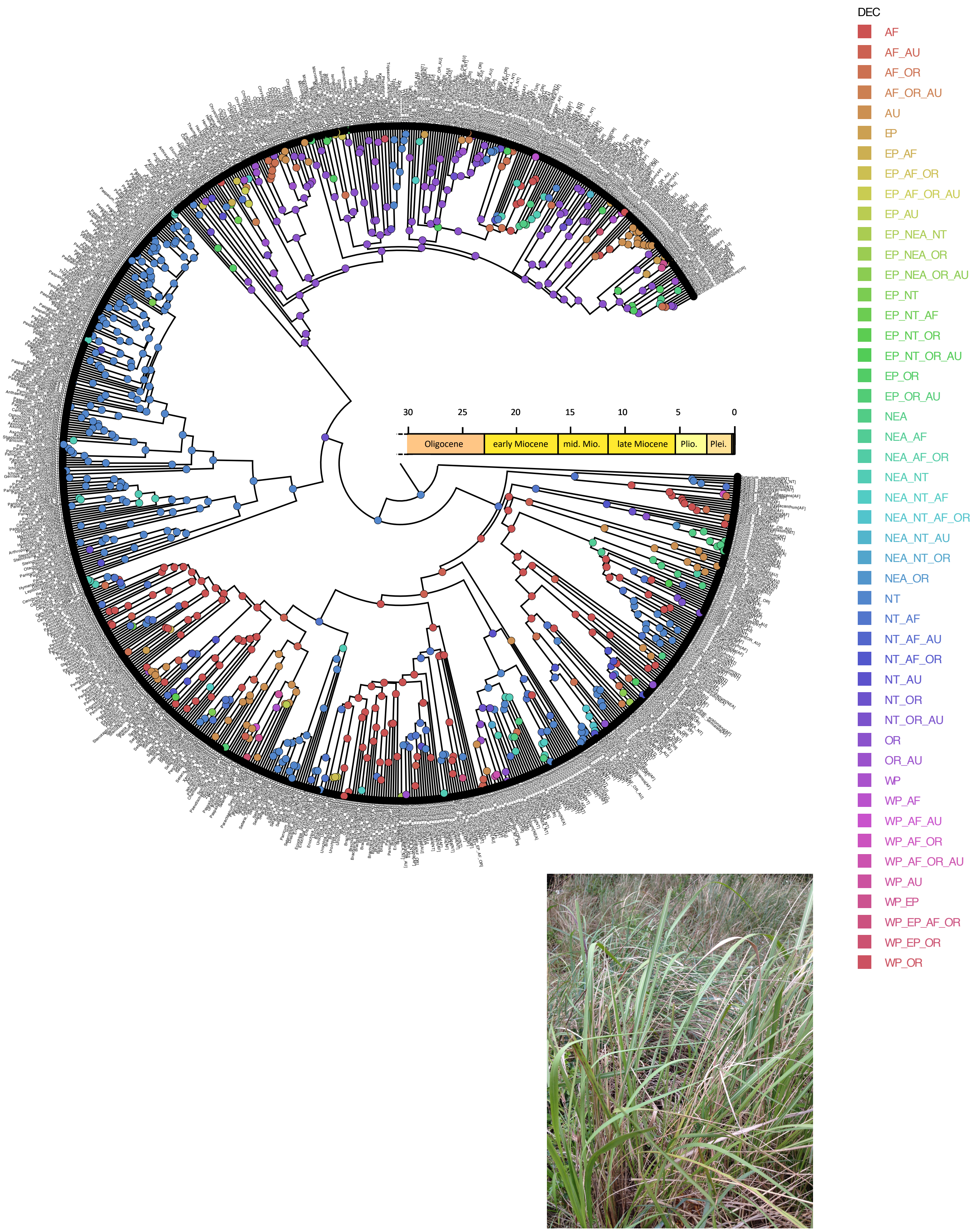

Supplementary Figure 7. Historical biogeography of Panicoideae, inferred using the DEC model. For each node, the most likely ancestral area (or combination of ancestral areas) is figured using a coloured circle. Major biogeographic areas are abbreviated as follow: Afrotropics (AF), Australasia (AU), East Palearctic (EP), Nearctic (NEA), Neotropics (NT), Oriental region (OR), and West Palearctic (WP). On the bottom right a picture of *Cymbopogon* sp. is presented for illustrative purpose. Copyright notes: the picture was taken by B. Le Ru (last author of the paper).

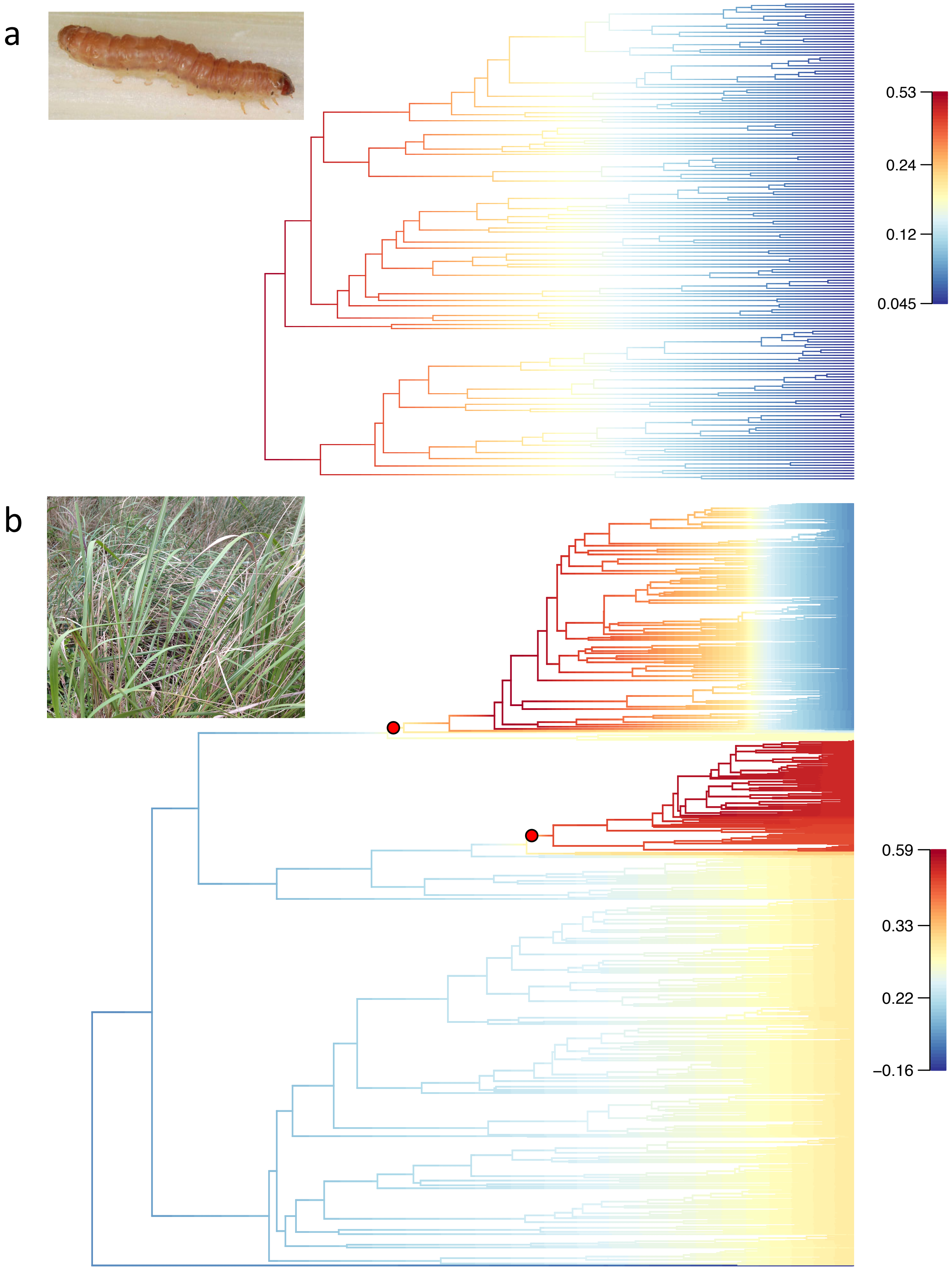

Supplementary Figure 8. BAMM analyses. Results of BAMM analyses relying on a model with a Poisson prior of 0.5 (see Supplementary Table S3 for more details). For all phylorate plots, speciation rates are indicated using a gradual scale (cool colours = slow; warm colours = fast). **(a)** On the top: phylorate plot for the Sesamiina dataset, which support a single diversification regime showing elevated speciation rates in the early stages of the insect lineage evolution, followed by a progressive slowdown but no drastic downward shift in speciation. **(b)** On the bottom: phylorate plot for the Panicoideae dataset, which support an opposite pattern with lower rates close to the origin of the group and increasing rates of speciation over time, accompanied by two significantly supported increases in speciation with the crown tribes Andropogoneae and Paspaleae (highlighted by red circles on the Figure). A picture of a larva of *Sesamia nonagrio*ides (Lefèbvre) (top left) and a picture of *Cymbopogon* sp. is presented for illustrative purpose. Copyright notes: all pictures were taken by B. Le Ru (last author of the paper).

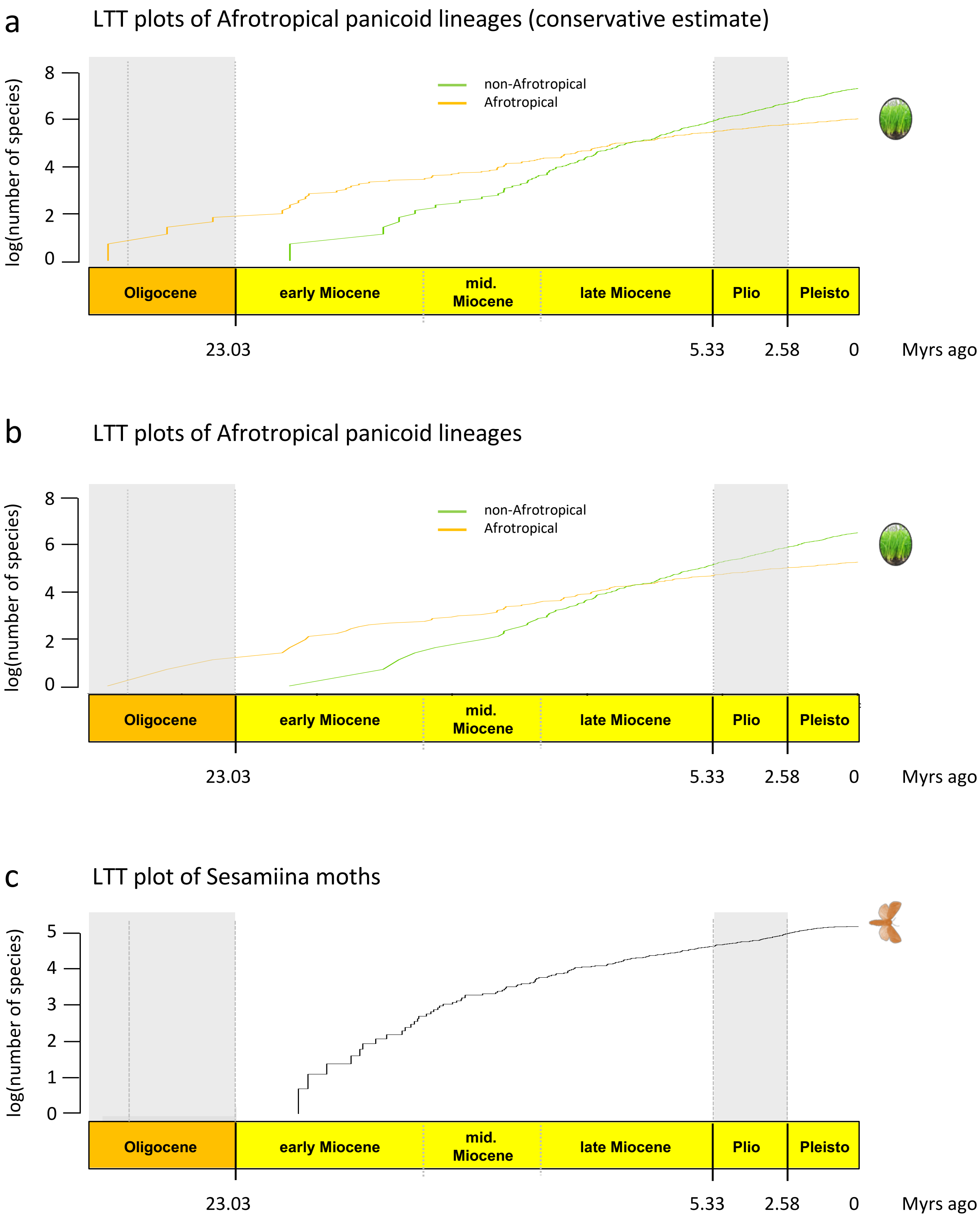

Supplementary Figure 9. Lineages-through-time plots for panicoid grasses and Sesamiina moths. **(a)** LTT plots for panicoid grasses, based on a timetree of 805 species; estimates for both Afrotropical and non-Afrotropical panicoid grass lineages rely on the most likely BiSSE model with a conservative estimate of 900 Afrotropical panicoid species. **(b)** LTT plots for panicoid grasses, based on a timetree of 805 species; estimates for both Afrotropical and non-Afrotropical panicoid grass lineages rely on the most likely BiSSE model with an estimate of 1,034 Afrotropical panicoid species. **(c)** LTT plot for the Sesamiina moths, based on a timetree of 181 species. Copyright notes: the grass and plant symbols rely on pictures taken by G.J. Kergoat and B. Le Ru (first and last authors of the study, respectively).

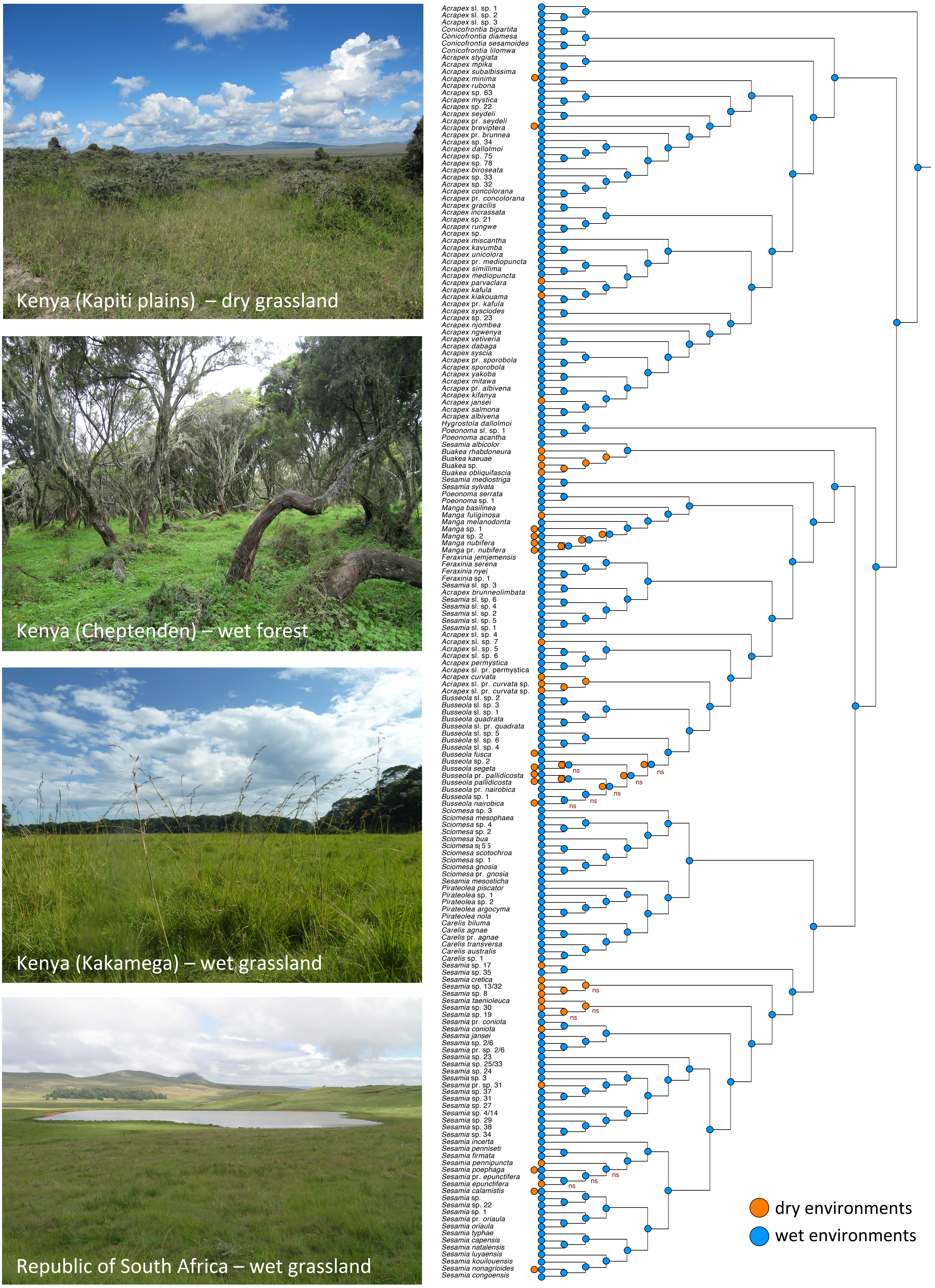

Supplementary Figure 10. Ancestral state estimations of ecological preferences. Results of the character optimization of ecological preferences, carried out under maximum likelihood with Mesquite. For each node, the most likely character state (or combination of character states) is figured; if the corresponding character state (or combination of character states) is not statistically supported the abbreviation 'ns' (for 'not supported') is added. Pictures of representative habitats are presented for illustrative purpose. Copyright notes: all pictures were taken by B. Le Ru (last author of the paper).

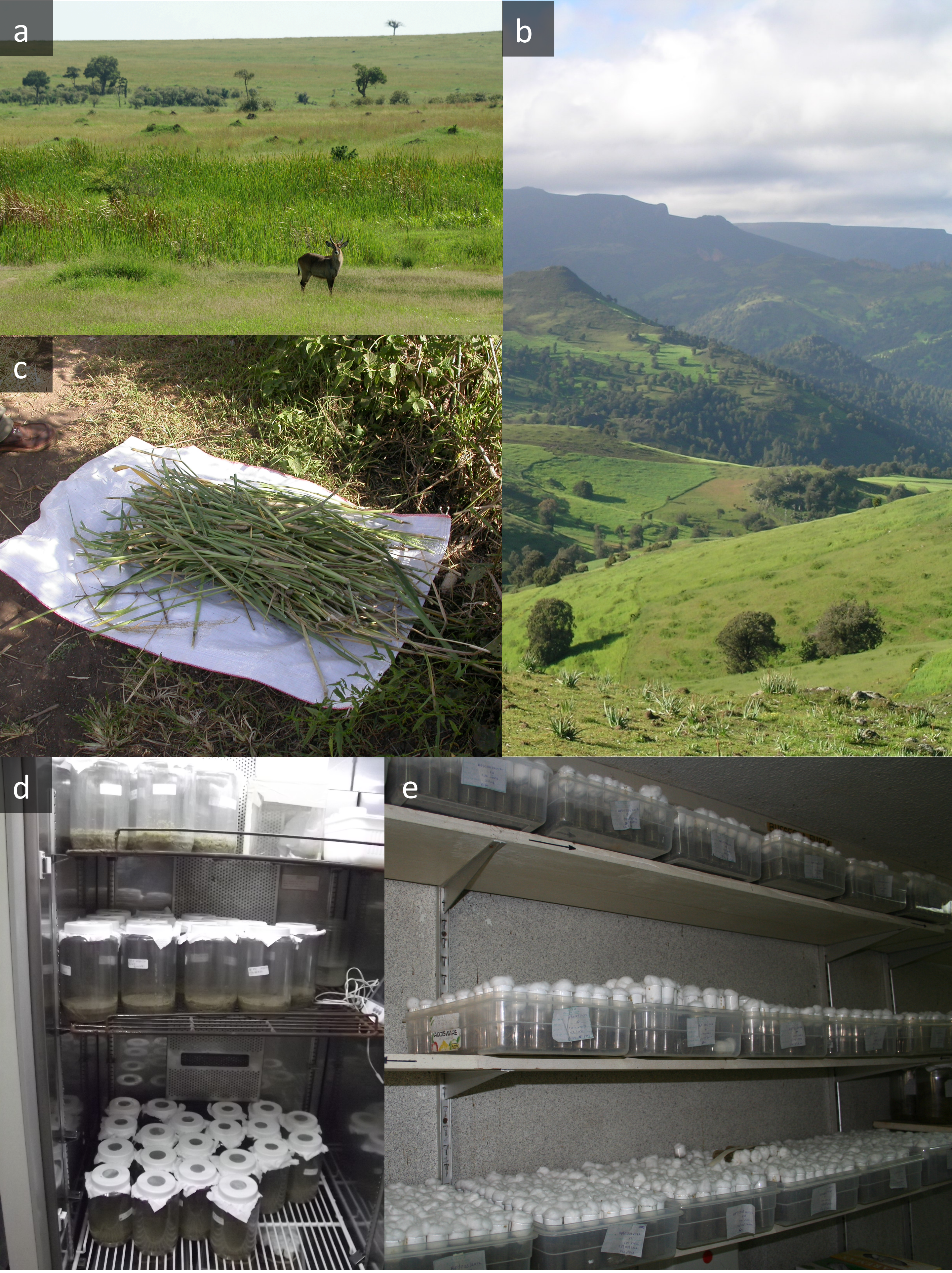

Supplementary Figure 11. Sampling of infested host-plants in the field following by the rearing of larvae on artificial diet. **(a)** Top left: typical open grassland habitat in Kenya. **(b)** Top right: open grasslands in the Ethiopian highlands. **(c)** Middle left: freshly cut stems of *Panicum maximum* Jacq. (Kenya). **(d)** & **(e)** Bottom: rearing rooms at *icipe* (Kenya). Copyright notes: all pictures were taken by B. Le Ru (last author of the paper).

Supplementary Note 1

Host-records

Based on the extant host-records listed above we have generated two graphs (see below) that underline the high level of host-specialization of Sesamiina stemborers. Based on our extant knowledge, most species are associated with three or less plant genera (94%) or species (88%).

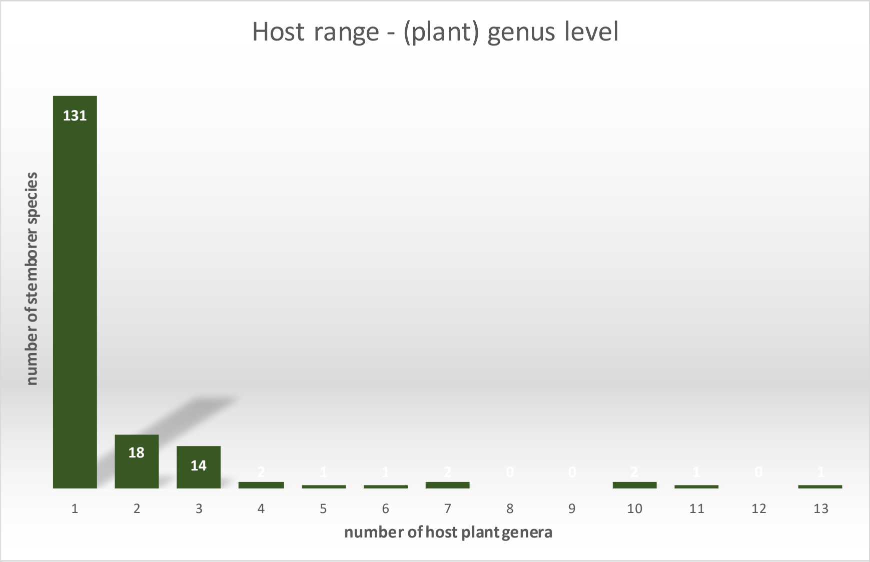

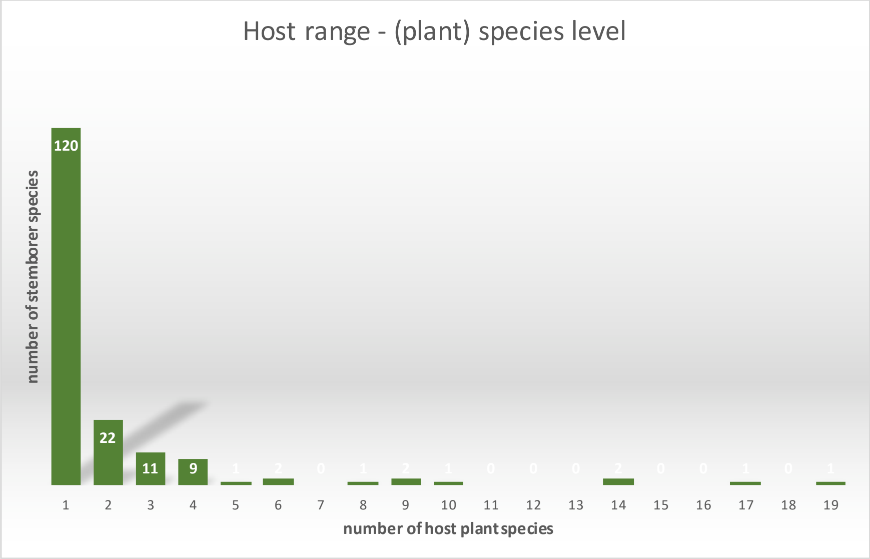

A few species are associated with a higher number of host-plant genera and species; although it is tempting to label them as polyphagous it is worth highlighting that they only feed on up to

three distinct plant families. The later has to be compared with truly polyphagous noctuid moths such as species in the genus *Spodoptera* that may feed on more than 25 plant families. Though future field studies on stemborers will likely unravel new host-plants and thus expand the host-range of several Sesamiina species, we think that the observed pattern of monophagy / oligophagy will remain still.

Ecological preferences and distribution

Sesamiina are unquestionably adapted to the main grassland habitat as attested by the fact that the great majority of stemborer species were collected with light-traps set in open habitats. Sesamiina stemborers also constitute one of the most abundant and conspicuous moth group in Afrotropical grasslands (Le Ru pers. obs.; based on more than 15 years of light trapping and grass sampling in Africa). The categorization made in Supplementary Data 2 reflects the preferences of species for contact zones between open grasslands and wetlands / marshes / wet forests. It is especially the case in low altitude areas; though most species exhibit preferences for wet habitats, a non-negligible portion of them (34 species; see above) are also adapted to drier conditions. It worth underlining that at mid-higher elevation stemborer species are only found in open grassland habitats, such as the Veld in South Africa or mountain grasslands in the East African mountain ranges. The later echoes that fact that the vast majority of Sesamiina species have a restricted geographic range, with species being generally associated with a specific bioregion or specific mountain ranges (e.g., Uzungwa mountains, Drakensberg range, Kipengere range). Species that supposedly had large distribution areas also generally correspond to species complexes.

Supplementary Note 2

We would like to thank several people for assistance with collection permits, field work and rearing of larvae: Michel Sezonlin and Georg Georgen for Benin, Casper Nyakumondiwa, Reyard Mutamisha and Eva Moeng for Botswana, Rose Ndemah and Philippe Le Gall for Cameroon, Onésime Mubenga Kandonda and Benjamin Dudu Akaibe for the Democratic Republic of Congo, Adugna Haile for Eritrea, Belay Defafachew and Mukuken Goftishu for Ethiopia, Boaz Musyoka, Leonard Ngala, Antony Kibe, Gerphas Okuku and George Ong'amo for Kenya, Jeannette Ravolonandrianina for Madagascar, Amelia Sidumo and Domingos Cugala for Mozambique, Zeena Abdullah and Abdallah Kidodi for Pemba and Zanzibar Islands, Grégoire Bani and Ange Kiakouama for the Republic of Congo, Desmond Conlong, Johnnie Van den Berg, Jurie Moolman, Mxolisi A. Stemele and Yoseph Assefa for the Republic of South Africa, Beatrice Pallangyo and Mohamedi Njaku for Tanzania, Agum Winnifred and Richard Molo for Uganda, Gilson Chipabika, Demian Mabote Ndalamei and Sylvia M. Tembo for Zambia. We are also grateful to the Boyekoli Ebale Congo Expedition, organized in 2010 by a consortium of three Belgian institutions – the Royal Museum of Central Africa, the Royal Belgian Institute of Natural Sciences and the National Botanical Garden of Belgium – in collaboration with the University of Kisangani (UNIKIS) in the Democratic Republic of the Congo, which allowed us to participate in the expedition and collect many interesting specimens. Finally, we also would like to thank the curators of NHM (Alberto Zilli and Martin Honey) and MCSN (Fabricio Rigato), MNHN (Joël Minet), MRAC (Ugo Dall'asta) and TMSA (Martin Krüger) for the permission to study and photograph the types.

Supplementary References

1. Wahlberg, N., Wheat, C. W. & Peña, C. Timing and patterns in the taxonomic diversification of Lepidoptera (butterflies and moths). *PLoS One* **8**, e80875 (2013).
2. Le Ru, B. P., Ong'amo, G. O., Moyal, P., Muchungu, E. & Ngala, L. Major ecological characteristics of East African noctuid stem borers. *Ann. Soc. Entomol. Fr.* **42**, 353–362 (2006a).
3. Le Ru, B. P., *et al.* Diversity of lepidopteran stem borers on monocotyledonous plants in eastern Africa and the islands of Madagascar and Zanzibar revisited. *Bull. Entomol. Res.* **96**, 555–563 (2006b).
4. Onyango, F. O. & Ochieng'Odero, J. P. R. Continuous rearing of the maize stem borer *Busseola fusca* on an artificial diet. *Entomol. Exp. Appl.* **73**, 139–144 (1994).
5. Le Ru, B. P., *et al.* Integrative taxonomy of *Acrapex* stem borers (Lepidoptera: Noctuidae: Apameini): combining morphology and Poisson Tree Process analyses. *Invertebr. Syst.* **28**, 451–475 (2014).
6. Le Ru, B. P., *et al.* Molecular phylogenetics and definition of the *Acrapex minima* Janse group (Lepidoptera, Noctuidae, Apameini, Sesamiina) with the description of four new species from the Afrotropics. *Ann. Soc. Entomol. Fr.* **53**, 219-235 (2017a).
7. Le Ru, B. P., *et al.* Phylogenetic analysis and systematics of the *Acrapex unicolora* Hampson species complex (Lepidoptera, Noctuidae, Noctuinae, Apameini) with the description of five news species from the Afrotropics. *Eur. J. Taxon.* **270**, 1–36 (2017b).
8. Le Ru, B. P., *et al.* Phylogeny and systematics of the *Acrapex apicestriata* (Bethune-Baker) species complex (Lepidoptera, Noctuidae, Noctuinae, Apameini) with the

description of eight new species from the Afrotropics. *Ann. Soc. Entomol. Fr.* **53**, 106–130 (2017c).

9. Le Ru, B. P., *et al.* A revision of the genus *Conicofrontia* Hampson (Lepidoptera: Noctuidae: Apameini), with description of a new species: new insights from morphological, ecological and molecular data. *Zootaxa* **3925**, 56–74 (2015).

10. Kergoat, G. J., *et al.* Integrative taxonomy reveals six new species related to the Mediterranean corn stalk borer *Sesamia nonagrioides* (Lefèbvre) (Lepidoptera, Noctuidae, Sesamiina). *Zool. J. Linn. Soc.* **175**, 244–270 (2015).

11. Kergoat, G. J., *et al.* Disentangling dispersal and vicariance patterns in armyworms: evolution and historical biogeography of the pest genus *Spodoptera* (Lepidoptera: Noctuidae). *Mol. Phylogenet. Evol.* **65**, 855–870 (2012).

12. Katoh, K. & Standley, D. M. MAFFT multiple sequence alignment software version 7: improvements in performance and usability. *Mol. Biol. Evol.* **30**, 772–780 (2013).

13. Maddison, W. P. & Maddison, D. R. Mesquite: a modular system for evolutionary analysis. Version 3.2 http://mesquiteproject.org (2017).

14. Nylander, J. A., Ronquist, F., Huelsenbeck, J. P. & Nieves-Aldrey, J. Bayesian phylogenetic analysis of combined data. *Syst. Biol.* **53**, 47–67 (2004).

15. Lanfear, R., Calcott, B., Ho, S. Y. & Guindon, S. PartitionFinder: combined selection of partitioning schemes and substitution models for phylogenetic analyses. *Mol. Biol. Evol.* **29**, 1695–1701 (2012).

16. Posada, D. & Buckley, T. R. Model selection and model averaging in phylogenetics: advantages of Akaike information criterion and Bayesian approaches over likelihood ratio tests. *Syst. Biol.* **53**, 793–808 (2004).

17. Stamatakis, A. RAxML version 8: a tool for phylogenetic analysis and post-analysis of large phylogenies. *Bioinformatics* **30**, 1312–1313 (2014).

18. Miller, M. A., *et al.* A RESTful API for access to phylogenetic tools via the CIPRES science gateway. *Evol. Bioinform. Online* **11**, 43 (2015).

19. Hillis, D. M. & Bull, J. J. An empirical test of bootstrapping as a method for assessing confidence in phylogenetic analysis. *Syst. Biol.* **42**, 182–192 (1993).

20. Nguyen, L. T., Schmidt, H. A., von Haeseler, A. & Minh, B. Q. IQ-TREE: a fast and effective stochastic algorithm for estimating maximum-likelihood phylogenies. *Mol. Biol. Evol.* **32**, 268–274 (2015).

21. Ronquist, F., *et al.* MrBayes 3.2: Efficient Bayesian phylogenetic inference and model choice across a large model space. *Syst. Biol.* **61**, 539–542 (2012).

22. Trifinopoulos, J., Nguyen, L. T., von Haeseler, A. & Minh, B. Q. W-IQ-TREE: a fast online phylogenetic tool for maximum likelihood analysis. *Nucleic Acids Res.* **44**, W232–W235 (2016).

23. Minh, B. Q., Nguyen, M. A. T. & von Haeseler, A. Ultrafast approximation for phylogenetic bootstrap. *Mol. Biol. Evol.* **30**, 1188–1195 (2013).

24. Ayres, D. L., *et al.* BEAGLE: an application programming interface and high-performance computing library for statistical phylogenetics. *Syst. Biol.* **61**, 170–173 (2012).

25. Erixon, P., Svennblad, B., Britton, T. & Oxelman, B. Reliability of Bayesian posterior probabilities and bootstrap frequencies in phylogenetics. *Syst. Biol.* **52**, 665–673 (2003).

26. Drummond, A. J., Suchard, M. A., Xie, D. & Rambaut, A. Bayesian phylogenetics with BEAUti and the BEAST 1.7. *Mol. Biol. Evol.* **29**, 1969–1973 (2012).

27. Drummond, A. J., Ho, S. Y., Phillips, M. J. & Rambaut, A. Relaxed phylogenetics and dating with confidence. *PLoS Biol.* **4**, e88 (2006).

28. Gernhard, T. The conditioned reconstructed process. *J. Theor. Biol.* **253**, 769–778 (2008).

29. Sohn, J. C., Labandeira, C., Davis, D. & Mitter, C. An annotated catalog of fossil and subfossil Lepidoptera (Insecta: Holometabola) of the world. *Zootaxa* **3286**, 132 (2012).

30. Mutanen, M., Wahlberg, N. & Kaila, L. Comprehensive gene and taxon coverage elucidates radiation patterns in moths and butterflies. *Proc. Biol. Sci. B* **277**, 2839–2848 (2010).

31. Toussaint, E. F. A., *et al.* Palaeoenvironmental shifts drove the adaptive radiation of a noctuid stemborer tribe (Lepidoptera, Noctuidae, Apameini) in the Miocene. *PLoS One* **7**, e41377 (2012).

32. Baele, G., *et al.* Improving the accuracy of demographic and molecular clock model comparison while accommodating phylogenetic uncertainty. *Mol. Biol. Evol.* **29**, 2157–2167 (2012).

33. Ree, R. H. & Smith, S. A. Maximum-likelihood inference of geographic range evolution by dispersal, local extinction, and cladogenesis. *Syst. Biol.* **57**, 4–14 (2008).

34. Smith, S. A. Taking into account phylogenetic and divergence-time uncertainty in a parametric biogeographical analysis of the Northern Hemisphere plant clade Caprifolieae. *J. Biogeogr.* **36**, 2324–2337 (2009).

35. Matzke, N. J. Model selection in historical biogeography reveals that founder-event speciation is a crucial process in island clades. *Syst. Biol.* **63**, 951–970 (2014).

36. Sanmartín, I. & Meseguer, A. S. Extinction in phylogenetics and biogeography: from timetrees to patterns of biotic assemblage. *Front. Genet.* **7**, 35 (2016).

37. Hobbie, E. A. & Werner, R. A. Intramolecular, compound-specific, and bulk carbon isotope patterns in C_3 and C_4 plants: a review and synthesis. *New Phytol.* **161**, 371–385 (2004).

38. Bruhl, J. J. & Wilson, K. L. Towards a comprehensive survey of C_3 and C_4 photosynthetic pathways in Cyperaceae. *Aliso* **23**, 99–148 (2007).

39. Edwards, E. J. & Smith, S. A. Phylogenetic analyses reveal the shady history of C_4 grasses. *Proc. Natl. Acad. Sci. USA* **107**, 2532–2537 (2010).

40. Taylor, S. H., *et al.* Ecophysiological traits in C_3 and C_4 grasses: a phylogenetically controlled screening experiment. *New Phytol.* **185**, 780–791 (2010).

41. Osborne, C. P., *et al.* A global database of C_4 photosynthesis in grasses. *New Phytol.* **204**, 441–446 (2014).

42. White, F. The vegetation of Africa, a descriptive memoir to accompany the UNESCO/AETFAT/UNSO vegetation map of Africa. *UNESCO Nat. Res.* **20**, 1–356 (1983).

43. Lewis, P. O. A likelihood approach to estimating phylogeny from discrete morphological character data. *Syst. Biol.* **50**, 913–925 (2001).

44. Schluter, D., Price, T., Mooers, A. Ø. & Ludwig, D. Likelihood of ancestor states in adaptive radiation. *Evolution* **51**, 1699–1711 (1997).

45. Spriggs, E. L., Christin, P. A. & Edwards, E. J. C_4 photosynthesis promoted species diversification during the Miocene grassland expansion. *PLoS One* **9**, e97722 (2014).

46. Prasad, V., *et al.* Late Cretaceous origin of the rice tribe provides evidence for early diversification in Poaceae. *Nat. Comm.* **2**, 480 (2011).

47. Christin, P. A., *et al.* Molecular dating, evolutionary rates, and the age of grasses. *Syst. Biol.* **63**, 153–165 (2014).

48. Mukherjee, S. K. Origin and distribution of *Saccharum*. *Int. J. Plant Sci.* **119**, 55–61 (1957).

49. Sauer, J. D. Revision of *Stenotaphrum* (Gramineae: Paniceae) with attention to its historical geography. *Brittonia* **24**, 202–222 (1972).

50. Yabuno, T. A biosystematic study on *Echinochloa oryzoides* (Ard.) Fritsch. *Cytologia* **49**, 673–678 (1984).

51. Raimondo, D., *et al. Red List of South African Plants. Strelitzia 25* (South African National Biodiversity Institute, Pretoria, 2009).

52. Wiersema, J. H. & León, B. *World Economic Plants: A Standard Reference, Second Edition* (CRS press, , Boca Raton, FL, 2013).

53. Hines, H. M. Historical biogeography, divergence times, and diversification patterns of bumble bees (Hymenoptera: Apidae: *Bombus*). *Syst. Biol.* **57**, 58–75 (2008).

54. Mansion, G., *et al.* Phylogenetic analysis informed by geological history supports multiple, sequential invasions of the Mediterranean Basin by the angiosperm family Araceae. *Syst. Biol.* **57**, 269–285 (2008).

55. Nylander, J. A., Olsson, U., Alström, P. & Sanmartín, I. Accounting for phylogenetic uncertainty in biogeography: a Bayesian approach to dispersal-vicariance analysis of the thrushes (Aves: *Turdus*). *Syst. Biol.* **57**, 257–268 (2008).

56. Tamme, R., *et al.* Predicting species' maximum dispersal distances from simple plant traits. *Ecology* **95**, 505-513 (2014).

57. Bouchenak-Khelladi, Y., Verboom, G. A., Savolainen, V. & Hodkinson T. R. Biogeography of the grasses (Poaceae): a phylogenetic approach to reveal evolutionary history in geographical space and geological time. *Bot. J. Linn. Soc.* **162**, 543–557 (2010).

58. Rabosky, D. L., *et al.* Rates of speciation and morphological evolution are correlated across the largest vertebrate radiation. *Nat. Comm.* **4**, 1958 (2013).

59. Morlon, H., *et al.* RPANDA: an R package for macroevolutionary analyses on phylogenetic trees. *Methods Ecol. Evol.* **7**, 589–597 (2016).

60. Stadler, T. Mammalian phylogeny reveals recent diversification rate shifts. *Proc. Natl. Acad. Sci. USA* **108**, 6187–6192 (2011).

61. Moore, B. R., Höhna, S., May, M. R., Rannala, B. & Huelsenbeck, J. P. Critically evaluating the theory and performance of Bayesian analysis of macroevolutionary mixtures. *Proc. Natl. Acad. Sci. USA* **113**, 9569–9574 (2016).

62. Rabosky, D. L., Mitchell, J. S. & Chang, J. Is BAMM flawed? Theoretical and practical concerns in the analysis of multi-rate diversification models. *Syst. Biol.* **66**, 477–498 (2017).

63. Rabosky, D. L., *et al.* BAMMtools: an R package for the analysis of evolutionary dynamics on phylogenetic trees. *Methods Ecol. Evol.* **5**, 701–707 (2014).

64. Morlon, H., Parsons, T. L. & Plotkin J. B. Reconciling molecular phylogenies with the fossil record. *Proc. Natl. Acad. Sci. USA* **108**, 16327–16332 (2011).

65. Posada, D. & Buckley, T. R. Model selection and model averaging in phylogenetics: advantages of Akaike information criterion and Bayesian approaches over likelihood ratio tests. *Syst. Biol.* **53**, 793–808 (2004).

66. Condamine, F. L., Rolland, J. & Morlon, H. Macroevolutionary perspectives to environmental change. *Ecol. Lett.* **16**, 72–85 (2013).

67. Zachos, J. C., Dickens, G. R. & Zeebe, R. E. An early Cenozoic perspective on greenhouse warming and carbon-cycle dynamics. *Nature* **451**, 279–283 (2008).

68. Beerling, D. J. & Royer, D. L. Convergent cenozoic CO_2 history. *Nat. Geosci.* **4**, 418–420 (2011).

69. Falkowski, P. G., *et al.* The rise of oxygen over the past 205 million years and the evolution of large placental mammals. *Science* **309**, 2202–2204 (2005).

70. Katz, M. E., *et al.* Biological overprint of the geological carbon cycle. *Marine Geol.* **217**, 323–338 (2005).

71. Erwin, D. H. Climate as a driver of evolutionary change. *Curr. Biol.* **19**, R575–R583 (2009).

72. Keeley, J. E. & Rundel, P. W. Fire and the Miocene expansion of C_4 grasslands. *Ecol. Lett.* **8**, 683–690 (2005).

73. Tipple, B. J. & Pagani, M. The early origins of terrestrial C_4 photosynthesis. *Annu. Rev. Earth Planet. Sci.* **35**, 435–461 (2007).

74. Hoetzel, S., Dupont, L., Schefuß, E., Rommerskirchen, F. & Wefer, G. The role of fire in Miocene to Pliocene C_4 grassland and ecosystem evolution. *Nat. Geosci.* **6**, 1027–1030 (2013).

75. Levin, N. E. Environment and climate of early human evolution. *Annu. Rev. Earth Planet. Sci.* **43**, 405–429 (2015).

76. Edwards, E. J., Osborne, C. O., Strömberg, C. A. E., Smith, S. A., & C_4 Grasses Consortium. The origins of C_4 grasslands: integrating evolutionary and ecosystem science. *Science* **328**, 587–591 (2010).

77. Uno, K. T., Polissar, P. J. & Jackson, K. E. Neogene biomarker record of vegetation change in eastern Africa. *Proc. Natl. Acad. Sci. USA* **113**, 6355–6363 (2016).

78. Etienne, R. S., *et al.* Diversity-dependence brings molecular phylogenies closer to agreement with the fossil record. *Proc. Biol. Sci. B* **279**, 1300–1309 (2012).

79. Maddison, W. P., Midford, P. E. & Otto, S. P. Estimating a binary character's effect on Speciation and extinction. *Syst. Biol.* **56**, 701–710 (2007).

80. Soreng, R. J. *et al.* A worldwide phylogenetic classification of the Poaceae (Gramineae). *J. Syst. Evol.* **53**, 117–137 (2015).
