# Supplementary material for: Opposite macroevolutionary responses to environmental changes in grasses and insects during the Neogene grassland expansion
Source: Nat Commun. 2018 Nov 30;9:5089. doi: 10.1038/s41467-018-07537-8 (PMC6269479; doi:10.1038/s41467-018-07537-8)
Supplement: Supplementary file 2 — Description of Additional Supplementary Files [file 41467_2018_7537_MOESM2_ESM.pdf]

Description of Additional Supplementary Files

File Name: Supplementary Data 1
Description: GenBank accession numbers.

File Name: Supplementary Data 2
Description: Known host-plant associations and ecological preferences for the sampled Sesamiina species.

File Name: Supplementary Data 3
Description: Coding of the panicoid species ranges for the historical biogeography analyses.
